# Future global urban water scarcity and potential solutions

**DOI:** 10.1038/s41467-021-25026-3

**Published:** 2021-08-03

**Authors:** Chunyang He, Zhifeng Liu, Jianguo Wu, Xinhao Pan, Zihang Fang, Jingwei Li, Brett A. Bryan

**Affiliations:** 1 Center for Human-Environment System Sustainability (CHESS), State Key Laboratory of Earth Surface Processes and Resource Ecology (ESPRE), Beijing Normal University, Beijing, China; 2 School of Natural Resources, Faculty of Geographical Science, Beijing Normal University, Beijing, China; 3 School of Life Sciences and School of Sustainability, Arizona State University, Tempe, AZ USA; 4 School of Environmental and Geographical Sciences (SEGS), Shanghai Normal University, Shanghai, China; 5 Centre for Integrative Ecology, Deakin University, Melbourne, Australia

**Keywords:** Environmental sciences, Geography, Water resources

## Abstract

Urbanization and climate change are together exacerbating water scarcity—where water demand exceeds availability—for the world’s cities. We quantify global urban water scarcity in 2016 and 2050 under four socioeconomic and climate change scenarios, and explored potential solutions. Here we show the global urban population facing water scarcity is projected to increase from 933 million (one third of global urban population) in 2016 to 1.693–2.373 billion people (one third to nearly half of global urban population) in 2050, with India projected to be most severely affected in terms of growth in water-scarce urban population (increase of 153–422 million people). The number of large cities exposed to water scarcity is projected to increase from 193 to 193–284, including 10–20 megacities. More than two thirds of water-scarce cities can relieve water scarcity by infrastructure investment, but the potentially significant environmental trade-offs associated with large-scale water scarcity solutions must be guarded against.

## Abstract (web summary)

This paper quantifies global urban water scarcity in 2016 and 2050 and explores potential solutions. One third to nearly half of the global urban population is projected to face water scarcity problems.

## Introduction

The world is rapidly urbanizing. From 1950 to 2020, the global population living in cities increased from 0.8 billion (29.6%) to 4.4 billion (56.2%) and is projected to reach 6.7 billion (68.4%) by 2050^1^. Water scarcity—where demand exceeds availability—is a key determinant of water security and directly affects the health and wellbeing of urban residents, urban environmental quality, and socioeconomic development^2–6^. At present, many of the world’s urban populations face water scarcity^3^. Population growth, urbanization, and socioeconomic development are expected to increase urban industrial and domestic water demand by 50–80% over the next three decades^4,7^. In parallel, climate change will affect the spatial distribution and timing of water availability^8,9^. As a result, urban water scarcity is likely to become much more serious in the future^10–12^, potentially compromising the achievement of the United Nations Sustainable Development Goals (SDGs) especially SDG11 *Sustainable Cities and Communities* and SDG6 *Clean Water and Sanitation*^13,14^.

Urban water scarcity has typically been addressed via engineering and infrastructure. Reservoirs are commonly used to store water during periods of excess availability and continuously supply water to cities to avoid water shortages during dry periods^15^. Desalination plants are increasingly used to solve water deficit problems for coastal cities^16^. For cities where local water resources cannot meet demand, inter-basin water transfer can also be an effective solution^17^ (Supplementary Table 8). However, investment in water infrastructure is costly; requires substantial human, energy, and material resources; is limited by natural conditions such as geographic location and topography; and may have very significant environmental impacts^2,3,18^. Hence, a comprehensive understanding of water scarcity and the potential solutions for the world’s cities is urgently required to promote more sustainable and livable urban futures^7,18,19^.

Previous studies have evaluated urban water scarcity^2,3,7,19^ (Supplementary Table 3). However, these studies have been limited in a number of ways including: assessing only a subset of the urban population (e.g., large cities only or regional in focus); considering only part of the water scarcity problem (i.e., availability but not withdrawal); or lacking a future perspective. For example, in assessing global urban water scarcity, Flörke et al.^7^ considered 482 cities (accounting for just 26% of the global urban population) under a business-as-usual scenario, and while McDonald et al.^2^ assessed a larger range of cities and scenarios, they considered water availability only, not withdrawals. As a result, significant uncertainty in estimates of current and future extent of urban water-scarcity remain, varying from 0.2 to 1 billion people affected in 2000 and from 0.5 to 4 billion in 2050 (Supplementary Table 4). A comprehensive assessment of global urban water scarcity is needed to identify cities at risk and provide better estimates of the number of people affected.

In addition, although many studies have discussed potential solutions to urban water scarcity, few have investigated the feasibility of these solutions for water-scarce cities at the global scale. Proposed solutions include groundwater exploitation, seawater desalination, increased water storage in reservoirs, inter-basin water transfer, improved water-use efficiency, and urban landscape management^2,3,14,19^. However, the potential effectiveness of these solutions for the world’s water-scarce cities depends on many factors including the severity of water scarcity, urban and regional geography and hydrogeology, socio-economic characteristics, and environmental carrying capacity^7,20^. Pairing the identification of water scarce cities with an evaluation of potential solutions is essential for guiding investment in future urban water security.

In this study, we comprehensively assessed global urban water scarcity in 2016 and 2050 and the feasibility of potential solutions for water-scarce cities. We first quantified the spatial patterns of the global urban population for 2016 at a grid-cell resolution of 1 km^2^ by integrating spatial urban land-use and population data. We then identified water-scarce areas at the catchment scale by combining global water resource availability and demand data, and calculated the global urban population in water-scarce areas in 2016. We also quantified the global urban population in water-scarce areas for 2050 under four socioeconomic and climate change scenarios by combining modeled projections of global urban area, population, and water availability and demand. Finally, we evaluated the feasibility of seven major solutions for easing water scarcity for each affected city. We discuss the implications of the results for mitigating global urban water scarcity and improving the sustainability and livability of the world’s cities.

## Results

### Current urban water scarcity

**Fig. 1:**
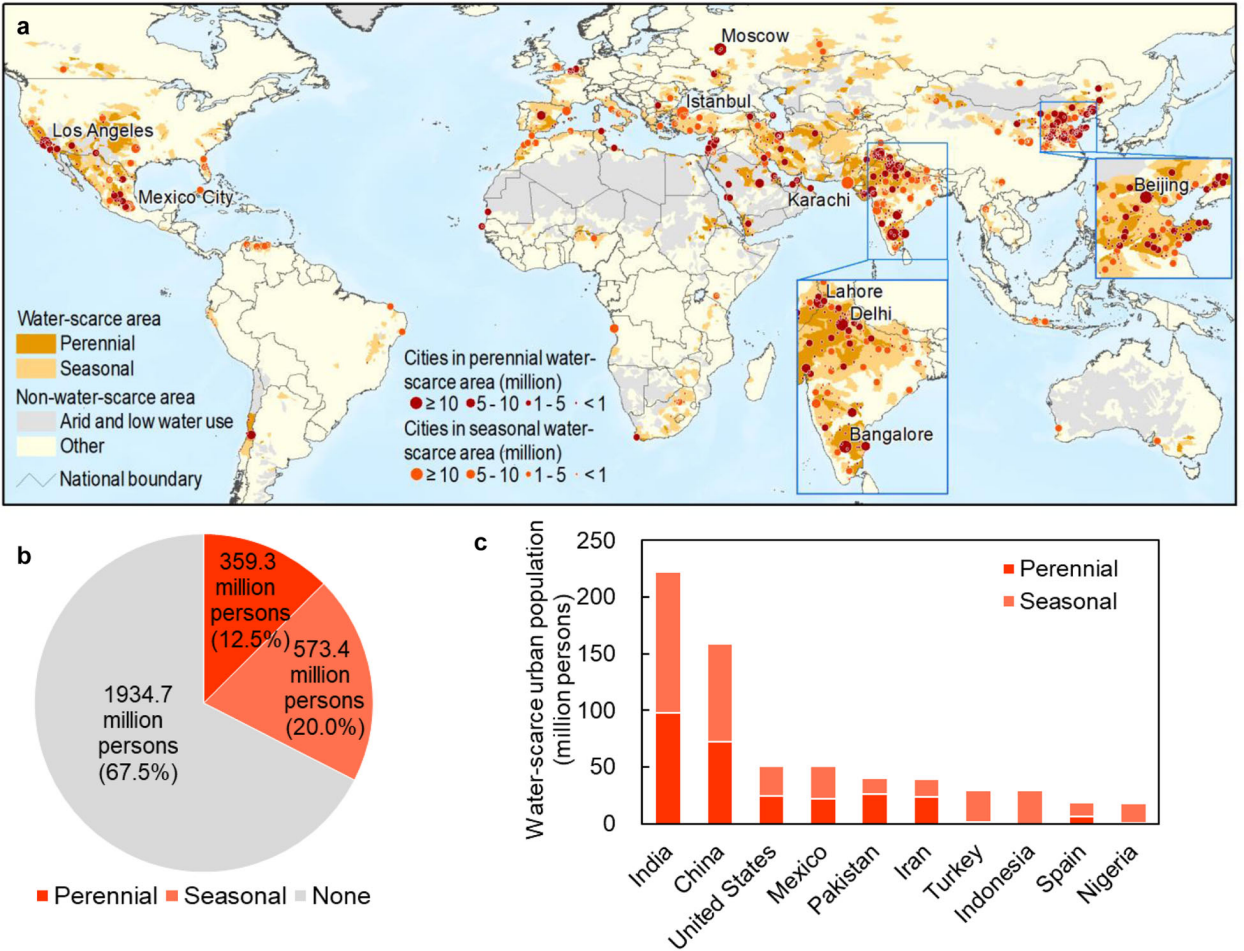
Current urban water scarcity. **a** spatial patterns of large cities in water-scarce areas (cities with population above 10 million in 2016 were labeled). **b** Water-scarce urban population at the global scale. **c** Water-scarce urban population at the national scale (10 countries with the largest values were listed). Please refer to Supplementary Data for urban water scarcity in each catchment.

Globally, 933 million (32.5%) urban residents lived in water-scarce regions in 2016 (Table 1, Fig. 1b) with 359 million (12.5%) and 573 million (20.0%) experiencing perennial and seasonal water scarcity, respectively. India (222 million) and China (159 million) had the highest urban populations facing water scarcity (Table 1, Fig. 1c).

**Table 1:** Global urban population facing water scarcity from 2016 to 2050 (million persons) including the 10 countries with the largest increases.

	2016			2050			Change between 2016 and 2050		
	Peren.	Seas.	Total	Peren.	Seas.	Total	Peren.	Seas.	Total
Asia	268.0	340.8	608.8	621.0 (374.3–711.4)	722.2 (495.0–968.0)	1343.2 (1090.2–1511.8)	131.7% (39.7%–165.4%)	111.9% (45.3%–184.1%)	120.6% (79.1%–148.3%)
India	98.0	124.1	222.1	255.1 (133.7–312.9)	295.3 (164.9–355.0)	550.4 (375.5–644.1)	160.4% (36.4%–219.4%)	137.9% (32.8%–186.0%)	147.8% (69.0%–190.0%)
China	72.1	86.9	158.9	128.7 (58.6–140.1)	112.2 (69.9–225.9)	241.0 (187.9–315.5)	78.7% (−18.7%–94.5%)	29.2% (−19.6%–160.1%)	51.6% (18.2%–98.6%)
Pakistan	25.7	14.2	39.9	70.8 (45.6–75.8)	26.7 (11.7–45.0)	97.5 (72.4–106.0)	175.9% (77.6%–195.2%)	87.4% (−18.1%–216.1%)	144.3% (81.5%–165.6%)
Indonesia	0.0	29.0	29.0	9.3 (0.0–38.9)	61.4 (28.0–100.7)	70.7 (30.2–107.9)		111.7% (−3.5%–247.5%)	143.7% (4.1%–272.3%)
Philippines	0.0	3.1	3.1	0.0 (0.0–4.1)	39.6 (5.5–51.5)	39.6 (5.5–51.5)		1164.3% (74.2%–1543.8%)	1164.3% (74.2%–1543.8%)
Africa	13.8	67.1	80.9	73.2 (33.3–115.1)	238.0 (133.3–338.9)	311.2 (210.3–372.2)	429.5% (140.5%–732.7%)	254.8% (98.7%–405.4%)	284.7% (159.9%–360.1%)
Nigeria	0.6	17.2	17.8	16.4 (0.0–30.5)	50.6 (22.2–76.9)	67.0 (40.4–85.1)	2741.1% (−100.0%–5196.9%)	194.2% (28.8%–346.8%)	276.7% (127.3%–378.6%)
Egypt	1.7	0.0	1.7	3.8 (3.3–4.2)	51.8 (0.0–57.9)	55.6 (3.6–61.6)	123.1% (91.1%–145.4%)		3154.9% (111.5%–3506.3%)
North America	45.7	62.8	108.5	89.9 (40.9–113.0)	103.6 (70.2–147.9)	193.5 (147.3–226.4)	97.0% (−10.4%–147.6%)	64.9% (11.7%–135.4%)	78.4% (35.8%–108.7%)
United States	24.0	26.9	50.9	41.8 (25.7–60.9)	50.7 (29.5–91.2)	92.6 (62.8–131.5)	74.0% (7.1%–153.5%)	88.6% (9.8%–239.0%)	81.7% (23.3%–158.2%)
Mexico	21.6	28.7	50.3	48.1 (9.8–66.4)	26.7 (15.4–58.7)	74.8 (62.9–89.9)	122.5% (−54.7%–207.2%)	–6.9% (−46.3%–104.3%)	48.6% (25.0%–78.5%)
South America	7.7	28.8	36.5	16.7 (8.7–41.8)	65.8 (43.9–130.4)	82.5 (52.7–160.9)	116.4% (13.4%–441.9%)	128.2% (52.4%–352.2%)	125.7% (44.2%–340.4%)
Brazil	0.1	7.7	7.7	1.6 (0.0–24.3)	33.7 (11.4–89.4)	35.3 (11.6–94.2)	2416.0% (−100.0%–38585.7%)	338.3% (48.9%–1063.3%)	355.1% (49.8%–1116.0%)
Europe	21.8	69.1	90.9	35.0 (14.4–42.6)	79.3 (66.0–132.9)	114.2 (84.5–162.2)	60.7% (−34.0%–96.0%)	14.7% (−4.5%–92.3%)	25.7% (−7.0%–78.5%)
Oceania	0.4	2.5	2.8	0.7 (0.1–2.6)	8.4 (2.6–14.8)	9.0 (3.0–15.4)	78.4% (−76.9%–608.1%)	241.9% (7.7%–502.6%)	220.4% (7.9%–444.1%)
World	359.3	573.4	932.7	839.6 (475.6–905.3)	1225.0 (902.3–1647.2)	2064.6 (1692.7–2373.0)	133.7% (32.4%–152.0%)	113.6% (57.4%–187.2%)	121.3% (81.5%–154.4%)

For 2050 the average values of urban population using the ensemble mean of runoff from GCMs under four scenarios are listed outside parentheses while the range (min-max) of estimations using runoff from each GCM under four scenarios are provided within parentheses.

Of the world’s 526 large cities (i.e., population >1 million), 193 (36.7%) were located in water-scarce regions (96 perennial, 97 seasonal) (Fig. 1a). Of the 30 megacities (i.e., population >10 million), 9 (30.0%) were located in water-scarce regions (Table 2). Six of these, including Los Angeles, Moscow, Lahore, Delhi, Bangalore, and Beijing, were located in regions with perennial water scarcity and three (Mexico City, Istanbul, and Karachi) were seasonally water-scarce (Fig. 1a).

**Table 2:** Megacities facing water scarcity from 2016 to 2050.

City	Country	Urban population in 2016 (thousand persons)	Water scarcity level				
			2016	2050			
				SSP1 & RCP2.6	SSP2 & RCP4.5	SSP3 & RCP7.0	SSP5 & RCP8.5
Delhi	India	26,720	Peren.	Peren.	Peren.	Peren.	Peren.
Shanghai	China	24,163	None	Seas.	Seas.	Peren.	Seas.
Mexico City	Mexico	21,420	Seas.	Seas.	Peren.	Peren.	Peren.
Sao Paulo	Brazil	21,136	None	None	Seas.	Seas.	None
Mumbai	India	19,535	None	None	Seas.	Seas.	Seas.
Cairo	Egypt	19,230	None	Seas.	Seas.	Seas.	Seas.
Beijing	China	18,812	Peren.	Peren.	Peren.	Peren.	Peren.
New York	United States	18,705	None	None	Seas.	None	None
Dhaka	Bangladesh	18,234	None	None	None	None	Seas.
Karachi	Pakistan	14,651	Seas.	Peren.	Peren.	Peren.	Seas.
Istanbul	Turkey	14,332	Seas.	Seas.	Seas.	Peren.	Peren.
Manila	Philippines	13,064	None	Seas.	Seas.	Seas.	Seas.
Tianjin	China	12,869	None	Seas.	None	Seas.	Seas.
Los Angeles	United States	12,383	Peren.	Peren.	Peren.	Peren.	Peren.
Moscow	Russia	12,168	Peren.	Peren.	Peren.	Peren.	Peren.
Lahore	Pakistan	10,808	Peren.	Peren.	Peren.	Peren.	Peren.
Bangalore	India	10,557	Peren.	Peren.	Peren.	Peren.	Peren.
Jakarta	Indonesia	10,287	None	None	None	Seas.	None
Lima	Peru	10,002	None	Seas.	Seas.	Seas.	Seas.
Number of perennial water-scarce megacities			6	7	8	10	8
Number of seasonal water-scarce megacities			3	7	8	7	8
Number of water-scarce megacities			9	14	16	17	16

### Urban water scarcity in 2050

**Fig. 2:**
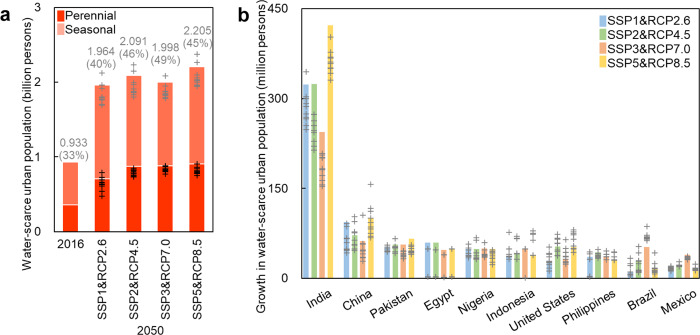
Changes in urban water scarcity from 2016 to 2050. **a** Changes in water-scarce urban population at the global scale. Bars present the simulated results using the ensemble mean of runoff from GCMs, the total values (i.e., perennial and seasonal), and percentages are labeled. Crosses (gray/black) present the simulated results (total/perennial) using runoff from each GCM. **b** Changes in water-scarce urban population at the national scale (10 countries with the largest values were listed). Bars present the total values simulated using the ensemble mean of runoff from GCMs. Crosses present the total values simulated using runoff from each GCM. Please refer to Supplementary Data for urban water scarcity in each catchment.

At the global scale, the urban population facing water scarcity was projected to increase rapidly, reaching 2.065 (1.693–2.373) billion people by 2050, a 121.3% (81.5–154.4%) increase from 2016 (Table 1, Fig. 2a). 840 (476–905) million people were projected to face perennial water scarcity and 1.225 (0.902–1.647) billion were projected to face seasonal water scarcity (Table 1). India’s urban population growth in water-scarce regions was projected to be much higher than other countries (Fig. 2b), increasing from 222 million people to 550 (376–644) million people in 2050 and accounting for 26.7% (19.2%–31.2%) of the world’s urban population facing water scarcity (Table 1).

**Fig. 3:**
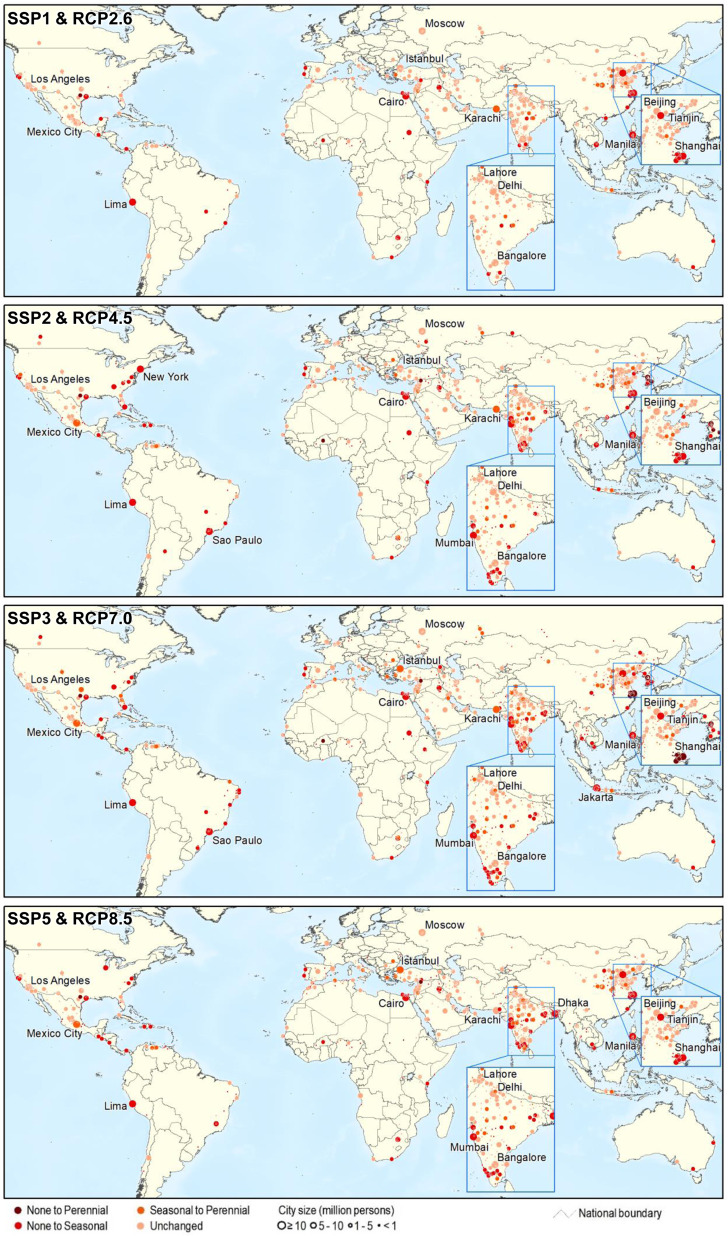
Changes in large cities subject to water scarcity from 2016 to 2050 under the four socio-economic and climate change scenarios. Only the water-scarce cities are listed. Cities with a population >10 million in 2016 are labeled.

Nearly half of the world’s large cities were projected to be located in water-scarce regions by 2050 (Fig. 3, Supplementary Fig. 3). The number of large cities facing water scarcity under at least one scenario was projected to increase to 292 (55.5%) by 2050. The number of megacities facing water scarcity under at least one scenario was projected to increase to 19 (63.3%) including 10 new megacities (i.e., Cairo, Dhaka, Jakarta, Lima, Manila, Mumbai, New York, Sao Paulo, Shanghai, and Tianjin) (Table 2).

### Factors influencing urban water scarcity

**Fig. 4:**
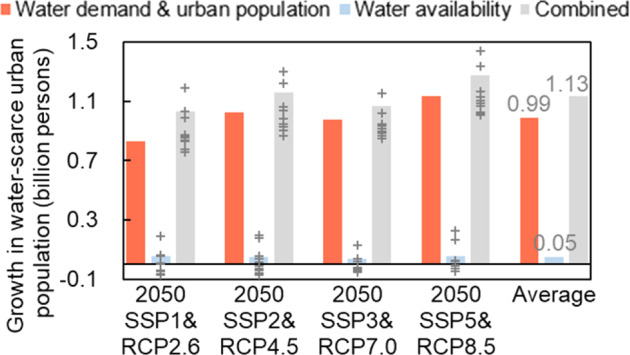
The effects of different factors on growth in global urban population exposed to water scarcity from 2016 to 2050. Bars present the simulated results using the ensemble mean of runoff from GCMs, crosses present the simulated results using runoff from each GCM.

Growth in urban population and water demand will be the main factor contributing to the increase in urban water scarcity (Fig. 4). From 2016 to 2050, population growth, urbanization, and socioeconomic development were projected to increase water demand and contribute to an additional 0.990 (0.829–1.135) billion people facing urban water scarcity, accounting for 87.5% (80.4–91.4%) of the total increase. Climate change was projected to alter water availability and increase the urban population subject to water scarcity by 52 (−72–229) million, accounting for 4.6% (−9.0–18.4%) of the total increase.

### Potential solutions to urban water scarcity

Water scarcity could be relieved for 276 (94.5%) large cities, including 17 (89.5%) megacities, via the measures assessed (Table 3, Supplementary Table 5). Among these, 260 (89.0%) cities have the option of implementing two or more measures. For example, Los Angeles can adopt desalination, groundwater exploitation, inter-basin water transfer, and/or virtual water trade (Table 3). However, 16 large cities, including two megacities (i.e., Delhi and Lahore) in India and Pakistan, are restricted by geography and economic development levels, making it difficult to adopt any of the potential water scarcity solutions (Table 3).

**Table 3:** Potential solutions for addressing water scarcity of large cities^*^.

City	Country	Feasibility of potential solutions							No solutions feasible
		SSP1&RCP2.6^a^	Desalination of sea water	Groundwater exploitation	Reservoir construction	Interbasin water transfer	Domestic virtual water trade	International water transfer/virtual water trade	
Delhi	India	○	○	○	○	○	○	○	∎
Shanghai	China	○	●	○	●	●	●	●	
Mexico City	Mexico	○	○	○	●	●	●	●	
Sao Paulo	Brazil	●	●	●	●	●	●	●	
Mumbai	India	●	●	●	●	○	○	○	
Cairo	Egypt	○	○	○	●	●	●	○	
Beijing	China	○	○	○	○	●	●	●	
New York	United States	●	●	●	●	●	●	●	
Dhaka	Bangladesh	●	●	○	○	●	●	○	
Karachi	Pakistan	○	●	●	○	○	○	○	
Istanbul	Turkey	○	●	●	●	●	●	●	
Manila	Philippines	○	●	●	●	●	●	○	
Tianjin	China	○	●	○	○	●	●	●	
Los Angeles	United States	○	●	●	○	●	●	●	
Moscow	Russia	○	○	●	○	●	●	●	
Lahore	Pakistan	○	○	○	○	○	○	○	∎
Bangalore	India	○	○	●	○	○	○	○	
Jakarta	Indonesia	●	●	●	●	●	●	○	
Lima	Peru	○	●	●	●	●	●	●	
Number of megacities^b^		5 (26.3%)	12 (63.2%)	11 (57.9%)	10 (52.6%)	14 (73.7%)	14 (73.7%)	10 (52.6%)	2 (10.5%)
Number of large cities^b^		68 (23.3%)	146 (50.0%)	192 (65.8%)	151 (51.7%)	200 (68.5%)	208 (71.2%)	190 (65.1%)	16 (5.5%)

*Cities with a population >10 million in 2016, which face water scarcity in 2050 under at least one scenario, were listed (sort by population from largest to smallest). Please refer to Supplementary Table 5 for all large cities. The black dots denote that the solution is applicable and probably can solve the issue, the white dots denote that the solution is inapplicable or cannot solve the issue, the black square denote that all the listed solutions are inapplicable or cannot solve the issue.

^a^Including improvement of water-use efficiency, limitation of population growth, and mitigation of climate change.

^b^Total number of water-scarce megacities/large cities, which could adopt the corresponding solution to solve water scarcity issue, was listed outside parentheses while the percentage of these cities to all water-scarce megacities/large cities was provided within parentheses.

Cities with a population >10 million in 2016, which face water scarcity in 2050 under at least one scenario, were listed (sort by population from largest to smallest)^*^. Please refer to Supplementary Table 5 for all large cities.

Domestic virtual water trade was the most effective solution, which could alleviate water scarcity for 208 (71.2%) large cities (including 14 (73.7%) megacities). Inter-basin water transfer could be effective for 200 (68.5%) large cities (including 14 (73.7%) megacities). Groundwater exploitation could be effective for 192 (65.8%) large cities (including 11 (57.9%) megacities). International water transfer and virtual water trade showed potential for 190 (65.1%) large cities (including 10 (52.6%) megacities). Reservoir construction could relieve water scarcity for 151 (51.7%) large cities (including 10 (52.6%) megacities). Seawater desalination has the potential to relieve water scarcity for 146 (50.0%) large cities (including 12 (63.2%) megacities). In addition, water scarcity for 68 (23.3%) large cities, including five megacities (i.e., New York, Sao Paulo, Mumbai, Dhaka, and Jakarta), could be solved via the water-use efficiency improvements, slowed population growth rate, and climate change mitigation measures considered under SSP1&RCP2.6.

## Discussion

We have provided a comprehensive evaluation of current and future global urban water scarcity and the feasibility of potential solutions for water-scarce cities. We found that the global urban population facing water scarcity was projected to double from 933 million (33%) in 2016 to 1.693–2.373 billion (35–51%) in 2050, and the number of large cities facing water scarcity under at least one scenario was projected to increase from 193 (37%) to 292 (56%). Among these cities, 276 large cities (95%) can address water scarcity through improving water-use efficiency, limiting population growth, and mitigating climate change under SSP1&RCP2.6; or via seawater desalination, groundwater exploitation, reservoir construction, interbasin water transfer, or virtual water trade. However, no solutions were available to relieve water scarcity for 16 large cities (5%), including two megacities (i.e., Delhi and Lahore) in India and Pakistan.

Previous studies have estimated the global urban population facing water scarcity to be between 150 and 810 million people in 2000, between 320 and 650 million people in 2010, and increasing to 0.479–1.445 billion people by 2050 (Supplementary Table 4). Our estimates of 933 million people in 2016 facing urban water scarcity, increasing to 1.693–2.373 billion people by 2050, are substantially higher than previously reported (Supplementary Fig. 5a). This difference is attributed to the fact that we evaluated the exposure of all urban dwellers rather than just those living in large cities (Supplementary Table 3). According to United Nations census data, 42% of the world’s urban population lives in small cities with a total population of <300,000 (Supplementary Fig. 4). Therefore, it is difficult to fully understand the global urban water scarcity only by evaluating the exposure of large cities. This study makes up for this deficiency and provides a comprehensive assessment of global urban water scarcity.

In addition, we used spatially corrected urban population data, newly released water demand/availability data, simulated runoff from GCMs in the most recent CMIP6 database, catchment-based estimation approach covering the upstream impacts on downstream water availability, and the new scenario framework combining socioeconomic development and climate change. Such data and methods can reduce the uncertainty in the spatial distribution of urban population and water demand/availability in the future, providing a more reliable assessment of global urban water scarcity.

Our projections suggest that global urban water scarcity will continue to intensify from 2016 to 2050 under all scenarios. By 2050, near half of the global urban population was projected to live in water-scarce regions (Figs. 2, 3). This will directly threaten the realization of SDG11 *Sustainable Cities and Communities* and SDG6 *Clean Water and Sanitation*. Although 95% of water-scarce cities can address the water crisis via improvement of water-use efficiency, seawater desalination, groundwater exploitation, reservoir construction, interbasin water transfer, or virtual water trade (Supplementary Table 5), these measures will not only have transformative impacts on society and the economy, but will also profoundly affect the natural environment. For example, the construction of reservoirs and inter-basin water transfer may cause irreversible damage to river ecosystems and hydrogeology and change the regional climate^4,15,17,21,22^. Desalination can have serious impacts on coastal zones and marine ecosystems^16,23^. Virtual water trade will affect regional economies, increase transport sector greenhouse gas emissions, and may exacerbate social inequality and affect the local environments where goods are produced^19,24^.

Water scarcity solutions may not be available to all cities. The improvement of water-use efficiency as well as other measures require the large-scale construction of water infrastructure, rapid development of new technologies, and large economic investment, which are difficult to achieve in low- and middle-income countries by 2050^14^. In addition, there will be 16 large cities, such as Delhi and Lahore, that cannot effectively solve the water scarcity problem via these measures (Supplementary Table 5). These cities also face several socioeconomic and environmental issues such as poverty, rapid population growth, and overextraction and pollution of groundwater^25,26^, which will further affect the achievement of SDG1 *No Poverty*, SDG3 *Good Health and Well-being*, SDG10 *Reduced Inequalities*, SDG14 *Life below Water* and SDG15 *Life on Land*.

To address global urban water scarcity and realize the SDGs, four directions are suggested. We need to:

Promote water conservation and reduce water demand. Our assessment provides evidence that the proposed water conservation efforts under SSP1&RCP2.6 are effective, which results in the least water-scarce urban population (34–241 million fewer compared to other SSPs&RCPs) at the global scale and can mitigate water scarcity for 68 (23.3%) large cities. The application of emerging water-saving technologies and the construction of sponge cities, smart cities, low-carbon cities, and resilient cities as well as the development of new theories and methods such as landscape sustainability science, watershed science, and geodesign will also play an important role for the further water demand reduction^5,6,27–29^. To implement these measures, the cooperation and efforts of scientists, policy makers and the public, as well as sufficient financial and material support are required. In addition, international cooperation must be strengthened in order to promote the development and dissemination of new technologies, assist in the construction of water infrastructure, and raise public awareness of water-savings, particularly in the Global South^30^.

Control population growth and urbanization in water-scarce regions by implementing relevant policies and regional planning. Urban population growth increases both water stress and the exposure of people, making it a key driver exacerbating global urban water scarcity^2^. Hence, the limitation of urban population growth in water-scarce areas can help to address this issue. According to our estimation, the control of urbanization under SSP3&RCP7.0, which has the lowest urbanization rate among four scenarios, can reduce the urban population subject to water scarcity by 93–207 million people compared with the business-as-usual scenario (SSP2&RCP4.5) and the rapid urbanization scenario (SSP5&RCP8.5), including 80–178 million people in India alone by 2050 (Fig. 2). To realize this pathway, policies that encourage family planning as well as tax incentives and regional planning for promoting population migration from water-scarce areas to other areas are needed^18^. In particular, for cities such as Delhi and Lahore that are both restricted by geography and socioeconomic disadvantage and have few options for dealing with water scarcity, there is an urgent need to control urban population growth and urbanization rates.

Mitigate climate change through energy efficiency and emissions abatement measures to avoid water resource impacts caused by the change in precipitation and the increase in evapotranspiration due to increased temperature. Our contribution analysis shows that the impacts of climate change on urban water scarcity is quite uncertain (ranging from a reduction of 72 million water-scarce urban people to an increase of 229 million) under different scenarios and GCMs (Fig. 4). On average, climate change under the business-as-usual scenario (SSP2&RCP4.5) will increase the global water-scarce urban population by 31 million in 2050. If the emissions reduction measures under SSP1&RCP2.6 are adopted, the increase in global water-scarce urban population due to climate change will be cut by half (16 million) in 2050. Thus, mitigating climate change is also important to reducing urban water scarcity. Considering that climate change in water-scarce areas would be affected by both internal and external impacts, mitigating climate change requires a global effort^31^.

Undertake integrated local sustainability assessment of water scarcity solutions. Our assessment reveals that 208 (71.2%) large cities may address water scarcity through seawater desalination, groundwater exploitation, reservoir construction, interbasin water transfer, and/or virtual water trade (Supplementary Table 5). While our results provide a guide at the global scale, city-level decisions about which measures to adopt to alleviate water scarcity involve very significant investments and should be supported by detailed local assessments of their relative effectiveness weighed against the potentially significant financial, environmental, and socio-economic costs. Integrated analyses are needed to quantify the effects of potential solutions on reducing water scarcity, their financial and resource requirements, and their potential impacts on socio-economic development for water-scarce cities and the sustainability of regional environments. To guard against the potential negative impacts of these measures, comprehensive impact assessments are required before implementing them, stringent regulatory oversight and continuous environmental monitoring are needed during and after their implementation, and policies and regulations should be established to achieve the sustainable supply and equitable distribution of water resources^24,32^.

Uncertainty is prevalent in our results due to limitations in the methodology and data used. First, constrained by data availability, in the evaluation of urban water scarcity in 2016 we used water demand/availability data for 2014 derived from the simulation results of the PCRGLOBWB 2 model, and only considered the inter-basin water transfers listed in City Water Map and the renewable groundwater simulated from the PCRGLOBWB 2 model instead of all available groundwater^3,33^. In the assessment of urban water scarcity and feasibility of potential solutions in 2050, we used water demand data derived from Hanasaki et al.^34^, in which irrigated area expansion, crop intensity change, and improvement in irrigation water efficiency were considered, but the change in irrigation to adapt to climate change as well as the impacts of energy systems (e.g., bio-energy production, mining, and fossil fuel extraction) on water demand were not fully considered^35^. Second, in order to maintain consistency and comparability of the water stress index (WSI) with the PCRGLOBWB 2 outputs^33^, environmental flow requirements were not considered. Following Mekonnen and Hoekstra^36^ and Veldkamp et al.^37^ (2017), we used an extreme threshold for WSI of 1.0 (where the entire water available is withdrawn for human use). If a more conservative threshold (e.g., WSI = 0.4 which is the threshold defining high water stress) was used, estimated global water scarcity and the urban population exposed to water stress would be much higher^7^.

In summary, global urban water scarcity is projected to intensify greatly from 2016 to 2050. By 2050, nearly half of the global urban population (1.693–2.373 billion) were projected to live in water-scarce regions, with about one quarter concentrated in India, and 19 (63%) global megacities are expected to face water scarcity. Increases in urban population and water demand drove this increase, while changes in water availability due to climate change compounded the problem. About 95% of all water-scarce cities could find at least one potential solution, but substantial investment is needed and solutions may have significant environmental and socioeconomic consequences. The aggravation of global urban water scarcity and the consequences of potential solutions will challenge the achievement of several SDGs. Therefore, there is an urgent need to further improve water-use efficiency, control urbanization in water-scarce areas, mitigate water availability decline due to climate change, and undertake integrated sustainability analyses of potential solutions to address urban water scarcity and promote sustainable development.

## Methods

### Description of scenarios used in this study

To assess future urban water scarcity, we used the scenario framework from the Scenario Model Intercomparison Project (ScenarioMIP), part of the International Coupled Model Intercomparison Project Phase 6 (CMIP6)^38^. The scenarios have been developed to better link the Shared Socioeconomic Pathways (SSPs) and Representative Concentration Pathways (RCPs) to support comprehensive research in different fields to better understand global climatic and socioeconomic interactions^38,39^. We selected the four ScenarioMIP Tier 1 scenarios (i.e., SSP1&RCP2.6, SSP2&RCP4.5, SSP3&RCP7.0, and SSP5&RCP8.5) to evaluate future urban water scarcity. SSP1&RCP2.6 represents the sustainable development pathway of low radiative forcing level, low climate change mitigation challenges, and low social vulnerability. SSP2&RCP4.5 represents the business-as-usual pathway of moderate radiative forcing and social vulnerability. SSP3&RCP7.0 represents a higher level of radiative forcing and high social vulnerability. SSP5&RCP8.5 represents a rapid development pathway and very high radiative forcing^38^.

### Estimation of urban water scarcity

To estimate urban water scarcity, we quantified the total urban population living in water-scarce areas^2,3,7,19^. Specifically, we first corrected the spatial distribution of the global urban population, then identified water-scarce areas around the world, and finally quantified the urban population in water-scarce areas at different scales (Supplementary Fig. 1).

#### Correcting the spatial distribution of global urban population

The existing global urban population data from the History Database of the Global Environment (HYDE) provided consistent information on historical and future population, but it has a coarse spatial resolution of 10 km (Supplementary Table 1)^40,41^. In addition, it was estimated using total population, urbanization levels, and urban population density, and does not align well with the actual distribution of urban land^42^. Hence, we allocated the HYDE global urban population data to high-resolution urban land data. We first obtained global urban land in 2016 from He et al.^42^. Since the scenarios used in existing urban land forecasts are now dated^43,44^, we simulated the spatial distribution of global urban land in 2050 under each SSP at a grid-cell resolution of 1km^2^ using the zoned Land Use Scenario Dynamics-urban (LUSD-urban) model^45–47^ (Supplementary Methods 1). The simulated urban expansion area in this study was significantly correlated with that in existing datasets (Supplementary Table 6). We then converted the global urban land raster layers for 2016 and 2050 into vector format to characterize the spatial extent of each city. The total population within each city was then summed and the remaining HYDE urban population cells located outside urban areas were allocated to the nearest city. Assuming that the population density within an urban area was homogeneous, we calculated the total population per square kilometer for all urban areas and converted this back to raster format at a spatial resolution of 1 km^2^. The new urban population data had much lower error than the original HYDE data (Supplementary Table 7).

#### Identification of global water-scarce areas

Annual and monthly WSI values were calculated at the catchment level in 2014 and 2050 as the ratio of water withdrawals (TWW) to availability (AWR)^33^. Due to limited data availability, we combined water-scarce areas in 2014 and the urban population in 2016 to estimate current urban water scarcity. WSI for catchment *i* for time *t* as:

1 $${{{{{\mathrm{WS{I}}}}}}}_{t,i}=\frac{{{{{\mathrm{TW{W}}}}}}_{t,i}}{{{{{\mathrm{AW{R}}}}}}_{t,i}}$$

For each catchment defined by Masutomi et al.^48^, the total water withdrawal (TWW_*t,i*_) equalled the sum of water withdrawals (WW_*t*,*n*,*i*_) for each sector *n* (irrigation, livestock, industrial, or domestic), while the water availability equalled the sum of available water resources for catchment *i* (*R*_*t*,*i*_), inflows/outflows of water resources due to interbasin water transfer ($\varDelta {{{{\mathrm{W{R}}}}}}_{t,i}$), and water resources from each upstream catchment *j* (WR_*t*,*i*,*j*_):

2 $${{{{{\mathrm{TW{W}}}}}}}_{t,i}={{\sum }_{n}{{{{\mathrm{WW}}}}}}_{t,n,i}$$

3 $${{{{{\mathrm{AW{R}}}}}}}_{t,i}={R}_{t,i}+\varDelta {{{{\mathrm{W{R}}}}}}_{t,i}+\mathop{\sum}\limits_{j}{{{{\mathrm{W{R}}}}}}_{t,i,j}$$

The changes of water resources due to interbasin water transfer were calculated based on City Water Map produced by McDonald et al.^3^. The number of water resources from upstream catchment *j* was calculated based on its water availability (AWR_*t*_,_*i*_,_*j*_) and water consumption for each sector *n* (WC_*t*,*n*,*i,j*_)^49^:

4 $${{{{{\mathrm{W{R}}}}}}}_{t,i,j}=\,\max (0,{{{{\mathrm{AW{R}}}}}}_{t,i,j}-{{\sum }_{n}{{{{\mathrm{WC}}}}}}_{t,n,i,j})$$

For areas without upstream catchments, the number of available water resources was equal to the runoff. Following Mekonnen and Hoekstra^36^, and Hofste et al.^33^, we did not consider environmental flow requirements in calculating water availability.

Annual and monthly WSI for 2014 were calculated directly based on water withdrawal, water consumption, and runoff data from AQUEDUCT3.0 (Supplementary Table 1). The data from AQUEDUCT3.0 were selected because they are publicly available and the PCRaster Global Water Balance (PCRGLOBWB 2) model used in the AQUADUCT 3.0 can better represent groundwater flow and available water resources in comparison with other global hydrologic models (e.g., the Water Global Assessment and Prognosis (WaterGAP) model)^33^. The annual and monthly WSI for 2050 were calculated by combining the global water withdrawal data from 2000 to 2050 provided by the National Institute of Environmental Research of Japan (NIER)^34^ and global runoff data from 2005 to 2050 from CMIP6 (Supplementary Table 1). Water withdrawal ${{{{{\mathrm{W{W}}}}}}}_{s,m,n,i}^{2050}$ in 2050 for each sector *n* (irrigation, industrial, or domestic), catchment *i*, and month *m* under scenario *s* was calculated based on water withdrawal in 2014 (${{{{{\mathrm{W{W}}}}}}}_{m,n,i}^{2014}$):

5 $${{{{{\mathrm{W{W}}}}}}}_{s,m,n,i}^{2050}={{{{\mathrm{W{W}}}}}}_{m,n,i}^{2014}\cdot [1+{{{{\mathrm{WW{R}}}}}}_{s,m,n,i}\cdot (2050-2014)]$$

adjusted by the mean annual change in water withdrawal from 2000 to 2050 (WWR_*s*, *m*, *n*, *i*_), calculated using the global water withdrawal for 2000 (${{{{{\mathrm{W{W}}}}}}}_{{{{{\mathrm{NIER}}}}},m,n,i}^{2000}$) and 2050 (${{{{{\mathrm{W{W}}}}}}}_{{{{{\mathrm{NIER}}}}},s,m,n,i}^{2050}$) provided by the NIER^34^:

6 $${{{{{\mathrm{WW{R}}}}}}}_{s,m,n,i}=\frac{({{{{\mathrm{W{W}}}}}}_{{{{{\mathrm{NIER}}}}},s,m,n,i}^{2050}/{{{{\mathrm{W{W}}}}}}_{{{{{\mathrm{NIER}}}}},m,n,i}^{2000})-1}{2050-2000}$$

Based on the assumption of a constant ratio of water consumption to water withdrawal in each catchment, water consumption in 2050 (${{{{{\mathrm{W{C}}}}}}}_{s,m,n,i}^{2050}$) was calculated as:

7 $${{{{{\mathrm{W{C}}}}}}}_{s,m,n,i}^{2050}={{{{\mathrm{W{W}}}}}}_{s,m,n,i}^{2050}\cdot \frac{{{{{\mathrm{W{C}}}}}}_{m,n,i}^{2014}}{{{{{\mathrm{W{W}}}}}}_{m,n,i}^{2014}}$$

where ${{{{{\mathrm{W{C}}}}}}}_{m,n,i}^{2014}$ denotes water consumption in 2014. Due to a lack of data, we specified that water withdrawal for livestock remained constant between 2014 and 2050, and used water withdrawal simulation under SSP3&RCP6.0 provided by the National Institute of Environmental Research in Japan to approximate SSP3&RCP7.0.

To estimate water availability, we calculated available water resources (${R}_{s,m,i}^{2041-2050}$) for each catchment *i* and month *m* under scenario *s* for the period of 2041–2050 as:

8 $${R}_{s,m,i}^{2041-2050}={R}_{m,i}^{{{{{\mathrm{ols}}}}},2005-2014}\cdot \frac{{\bar{R}}_{s,m,i}^{2041-2050}}{{\bar{R}}_{m,i}^{2005-2014}}$$

based on the amount of available water resources with 10-year ordinary least square regression from 2005 to 2014 (${R}_{m,i}^{{{{{\mathrm{ols}}}}},\,2005-2014}$) from AQUEDUCT3.0 (Supplementary Table 1). ${\overline{R}}_{m,i}^{2005-2014}$ and ${\overline{R}}_{s,m,i}^{2041-2050}$ denote the multi-year average of runoff (i.e., surface and subsurface) from 2005 to 2014, and from 2041 to 2050, respectively, calculated using the average values of simulation results from 10 global climate models (GCMs) (Supplementary Table 2).

We then identified water-scarce catchments based on the WSI. Two thresholds of 0.4 and 1.0 have been used to identify water-scarce areas from WSI (Supplementary Table 4). While the 0.4 threshold indicates high water stress^49^, the threshold of 1.0 has a clearer physical meaning, i.e., that water demand is equal to the available water supply and environmental flow requirements are not met^36,37^. We adopted the value of 1.0 as a threshold representing extreme water stress to identify water-scarce areas. The catchments with annual WSI >1.0 were identified as perennial water-scarce catchments; the catchments with annual WSI equal to or <1.0 and WSI for at least one month >1.0 were identified as seasonal water-scarce catchments.

#### Estimation of global urban water scarcity

Based on the corrected global urban population data and the identified water-scarce areas, we evaluated urban water scarcity at the global and national scales via a spatial overlay analysis. The urban population exposed to water scarcity in a region (e.g., the whole world or a single country) is equal to the sum of the urban population in perennial water-scarce areas and that in seasonal water-scarce areas. Limited by data availability, we used water-scarce areas in 2014 and the urban population in 2016 to estimate current urban water scarcity. Projected water-scarce areas and urban population in 2050 under four scenarios were then used to estimate future urban water scarcity. In addition, we obtained the location information of large cities (with population >1 million in 2016) from the United Nations’ World Urbanization Prospects^1^ (Supplementary Table 1) and identified those in perennial and seasonal water-scarce areas.

#### Uncertainty analysis

To evaluate the uncertainty across the 10 GCMs used in this study (Supplementary Table 2), we identified water-scarce areas and estimated urban water scarcity using the simulated runoff from each GCM under four scenarios. To perform the uncertainty analysis, the runoff in 2050 for each GCM was calculated using the following equation:

9 $${R}_{s,g,m,i}^{2050}={R}_{m,i}^{2014}\cdot \frac{{R}_{s,g,m,i}^{2041-2050}}{{R}_{g,m,i}^{2005-2014}}$$

where ${R}_{s,g,m,i}^{2050}$ denotes the runoff of catchment *i* in month *m* in 2050 for GCM *g* under scenario *s*. ${R}_{g,m,i}^{2005-2014}$ and ${R}_{s,g,m,i}^{2041-2050}$ denote the multi-year average runoff from 2005 to 2014, and from 2041 to 2050, respectively, calculated using the simulation results from GCM *g*. Using the runoff for each GCM, the WSI in 2050 for each catchment was recalculated, water-scarce areas were identified, and the urban population exposed to water scarcity was estimated.

#### Contribution analysis

Based on the approach used by McDonald et al.^2^ and Munia et al.^50^, we quantified the contribution of socioeconomic factors (i.e., water demand and urban population) and climatic factors (i.e., water availability) to the changes in global urban water scarcity from 2016 to 2050. To assess the contribution of socioeconomic factors (${{{{{\mathrm{Co{n}}}}}}}_{s,{{{{\mathrm{SE}}}}}}$), we calculated global urban water scarcity in 2050 while varying demand and population and holding catchment runoff constant (${{{{{\mathrm{UW{S}}}}}}}_{s,{{{{\mathrm{SE}}}}}}^{2050}$). Conversely, to assess the contribution of climate change ($Co{n}_{s,CC}$), we calculated scarcity while varying runoff and holding urban population and water demand constant (${{{{{\mathrm{UW{S}}}}}}}_{s,{{{{\mathrm{CC}}}}}}^{2050}$). Socioeconomic and climatic contributions were then calculated as:

10 $${{{{{\mathrm{Co{n}}}}}}}_{s,SE}=\frac{{{{{\mathrm{UW{S}}}}}}_{s,{{{{\mathrm{SE}}}}}}^{2050}-{{{{\mathrm{UW{S}}}}}}^{2016}}{{{{{\mathrm{UW{S}}}}}}_{s}^{2050}-{{{{\mathrm{UW{S}}}}}}^{2016}}\times 100 \%$$

11 $${{{{{\mathrm{Co{n}}}}}}}_{s,CC}=\frac{{{{{\mathrm{UW{S}}}}}}_{s,{{{{\mathrm{CC}}}}}}^{2050}-{{{{\mathrm{UW{S}}}}}}^{2016}}{{{{{\mathrm{UW{S}}}}}}_{s}^{2050}-{{{{\mathrm{UW{S}}}}}}^{2016}}\times 100 \%$$

### Feasibility analysis of potential solutions to urban water scarcity

Potential solutions to urban water scarcity involve two aspects: increasing water availability and reducing water demand^2^. Approaches to increasing water availability include groundwater exploitation, seawater desalination, reservoir construction, and inter-basin water transfer; while approaches to reduce water demand include water-use efficiency measures (e.g., new cultivars for improving agricultural water productivity, sprinkler or drip irrigation for improving water-use efficiency, water-recycling facilities for improving domestic and industrial water-use intensity), limiting population growth, and virtual water trade^2,3,18,32^. To find the best ways to address urban water scarcity, we assessed the feasibility of these potential solutions for each large city (Supplementary Fig. 2).

First, we divided these solutions into seven groups according to scenario settings and the scale of implementation of each solution (Supplementary Fig. 2). Among the solutions assessed, water-use efficiency improvement, limiting population growth, and climate change mitigation were included in the simulation of water demand and water availability under the ScenarioMIP SSPs&RCPs simulations^34^. Here, we considered the measures within SSP1&RCP2.6 which included the lowest growth in population, irrigated area, crop intensity, and greenhouse gas emissions; and the largest improvements in irrigation, industrial, and municipal water-use efficiency^34^.

We then evaluated the feasibility of the seven groups of solutions according to the characteristics of water-scarce cities (Supplementary Fig. 2). Of the 526 large cities (with population >1 million in 2016 according to the United Nations’ World Urbanization Prospects), we identified those facing perennial or seasonal water scarcity under at least one scenario by 2050. We then selected the cities that no longer faced water scarcity under SSP1&RCP2.6 where the internal scenario assumptions around water-use efficiency, population growth, and climate change were sufficient to mitigate water scarcity. Following McDonald et al.^2,3^ and Wada et al.^18^, we assumed that desalination can be a potential solution for coastal cities (distance from coastline <100 km) and groundwater exploitation can be feasible for cities where the groundwater table has not significantly declined. For cities in catchments facing seasonal water scarcity and with suitable topography, reservoir construction was identified as a potential solution. Inter-basin water transfer was identified as a potential solution for a city if nearby basins (i.e., in the same country, <1000 km away [the distance of the longest water transfer project in the world]) were not subject to water scarcity and had sufficient water resources to address the water scarcity for the city. Domestic virtual water trade was identified as a potential solution for a city if it was located in a country without national scale water scarcity. International water transfer or virtual water trade was identified as a feasible solution for cities in middle and high-income countries. Based on the above assumptions, we identified potential solutions to water scarcity in each city (see Supplementary Table 1 for the data used).

## Supplementary information

Supplementary Information

## Data Availability

All the data created in this study are openly available and the download information of supplementary data can be found in Github repositories with the identifier https://github.com/zfliu-bnu/Urban-water-scarcity. Other data are available from the corresponding author upon reasonable request.
