# Supplementary material for: Future global urban water scarcity and potential solutions
Source: Nat Commun. 2021 Aug 3;12:4667. doi: 10.1038/s41467-021-25026-3 (PMC8333427; doi:10.1038/s41467-021-25026-3)
Supplement: Supplementary file 1 — Supplementary Information [file 41467_2021_25026_MOESM1_ESM.pdf]

Supplementary Information

Future global urban water scarcity and potential solutions

Chunyang He[1,2], Zhifeng Liu[1,2*], Jianguo Wu[1,2,3], Xinhao Pan[1,2], Zihang Fang[1,2], Jingwei Li[4], Brett A Bryan[5]

1 Center for Human-Environment System Sustainability (CHESS), State Key Laboratory of Earth Surface Processes and Resource Ecology (ESPRE), Beijing Normal University, Beijing, 100875, China

2 School of Natural Resources, Faculty of Geographical Science, Beijing Normal University, Beijing, 100875, China

3 School of Life Sciences and School of Sustainability, Arizona State University, Tempe, AZ 85287, USA

4 School of Environmental and Geographical Sciences (SEGS), Shanghai Normal University, Shanghai, 200234, China

5 Centre for Integrative Ecology, Deakin University, Melbourne, VIC3125, Australia

Contents

Supplementary Methods 1. The simulation of global urban land in 2050.

Supplementary Figure 1. Flow chart for estimating urban water scarcity.

Supplementary Figure 2. Framework for selecting potential solutions.

Supplementary Figure 3. Large cities subject to water scarcity in 2050 under four socio-economic and climate change scenarios.

Supplementary Figure 4. Distribution of urban population in different city sizes in 2016.

Supplementary Figure 5. Comparing urban exposure to water scarcity between our study and previous studies.

Supplementary Table 1. Details of the data sources used in this study.

Supplementary Table 2. Details of the global climate models used in this study.

Supplementary Table 3. Details of this study and comparison with previous studies.

Supplementary Table 4. Results of this study and comparison with previous studies.

Supplementary Table 5. Potential solutions for addressing water scarcity of different cities.

Supplementary Table 6. Spatial correlation on future urban expansion area between our estimates and existing datasets.

Supplementary Table 7. Comparison of urban population in cities with different sizes in 2016.

Supplementary Table 8. Impacts of interbasin water transfer on urban population facing water scarcity in 2016.

Supplementary Methods 1. The simulation of global urban land in 2050.

Considering the obvious differences in the natural environments and socioeconomic characteristics in different regions across the world, we applied the zonal simulation approach used by Liu et al. (2019), Chen et al. (2020) and Gao and O'Neill (2020) and the Land Use Scenario Dynamics-urban (LUSD-urban) model to simulate the spatial pattern of global urban land in 2050. The model calculates urban land demand and spatially allocates urban land based on the principle of urban land supply-demand balance (He et al., 2008; 2015).

First, we divided the world into 100 km × 100 km grid cells to reflect the differences in urban expansion due to diverse socioeconomic and natural characteristics in different regions. In each grid cell, we obtained the total urban population for 1992-2016 from History Database of the Global Environment (HYDE) and the urban land area for 1992-2016 from He et al. (2019) (Supplementary Table 1), and then constructed a linear regression model using urban land area as the dependent variable and urban population as the independent variable:

$$UL_{t,i} = a_i \cdot UP_{t,i} + b_i \tag{1}$$

where $UL_{t,i}$ and $UP_{t,i}$ denote the urban land area and the number of urban population in the *i*-th grid cell in year *t*, respectively. a_i and b_i denote the slope and intercept, respectively.

Then, we calculated the urban land demand in each grid cell based on the linear regression model and urban population data from HYDE in 2050 under different scenarios (Supplementary Table 1). Based on the urban land demand, we used the LUSD-urban model to simulate the spatial allocation of urban land at a 1-km resolution in each grid cell. Specifically, we calculated the probability of all non-urban pixels to be converted to urban pixels in each grid cell. The calculation process can be expressed as:

$$P_{t,k,i,j} = \left(\sum_{n=1}^{m-2} W_n \cdot S_{t,n,i,j} + W_{m-1} \cdot N_{t,i,j} - W_m \cdot I_{t,k,i,j} \right) \cdot \prod_{r=1} EC_{t,r,i,j} \cdot V_{t,i,j} \tag{2}$$

where $P_{t,k,i,j}$ denotes the probability that each non-urban pixel *j* with land cover type *k* in grid *i* will be converted to an urban pixel in year *t*. $S_{t,n,i,j}$ denotes the normalized score of the suitability factor *n*. W_n denotes the weight of the suitability factor *n*. The suitability factors used in this study include elevation, slope, distance to cities with different size (with populations above 10 million, between 5-10 million, between 3-5 million, and between 1-3 million), distance to coastlines, distance to railways and roads, and river density (Supplementary Table 1). $N_{t,i,j}$ denotes the neighborhood effects, W_{m-1} denotes the weight of the neighborhood effects. $I_{t,k,i,j}$ denotes the inheritance effects, W_m denotes the weight of the inheritance effects. $EC_{t,r,i,j}$ denotes the ecological restriction, and all the pixels in the protected area have the $EC_{t,r,i,j}$ value of 0. $V_{t,i,j}$ denotes the random factor, which can be expressed as:

$$V_{t,i,j} = 1 + [-\ln(rand)]^a \tag{3}$$

where *rand* denotes a random variable whose value ranges from 0 to 1, and conforms to a uniform distribution. *a* denotes an adjustment factor that controls the degree of random disturbance. Based on He et al. (2008; 2015), the Monte Carlo method was used to calibrate the weights of each grid. Accuracy assessment showed that the simulated global urban land in 2016 had a Kappa coefficient of 0.60, indicating that the zoned LUSD-urban model projections were of sufficient accuracy to simulate the spatial patterns of global urban land. Based on the calibrated model, we simulated the urban expansion in each grid cell from 2016 to 2050. Finally, we obtained the global urban land data, at a spatial resolution of 1 km, in 2050 under different scenarios by integrating the simulation results of all grids.

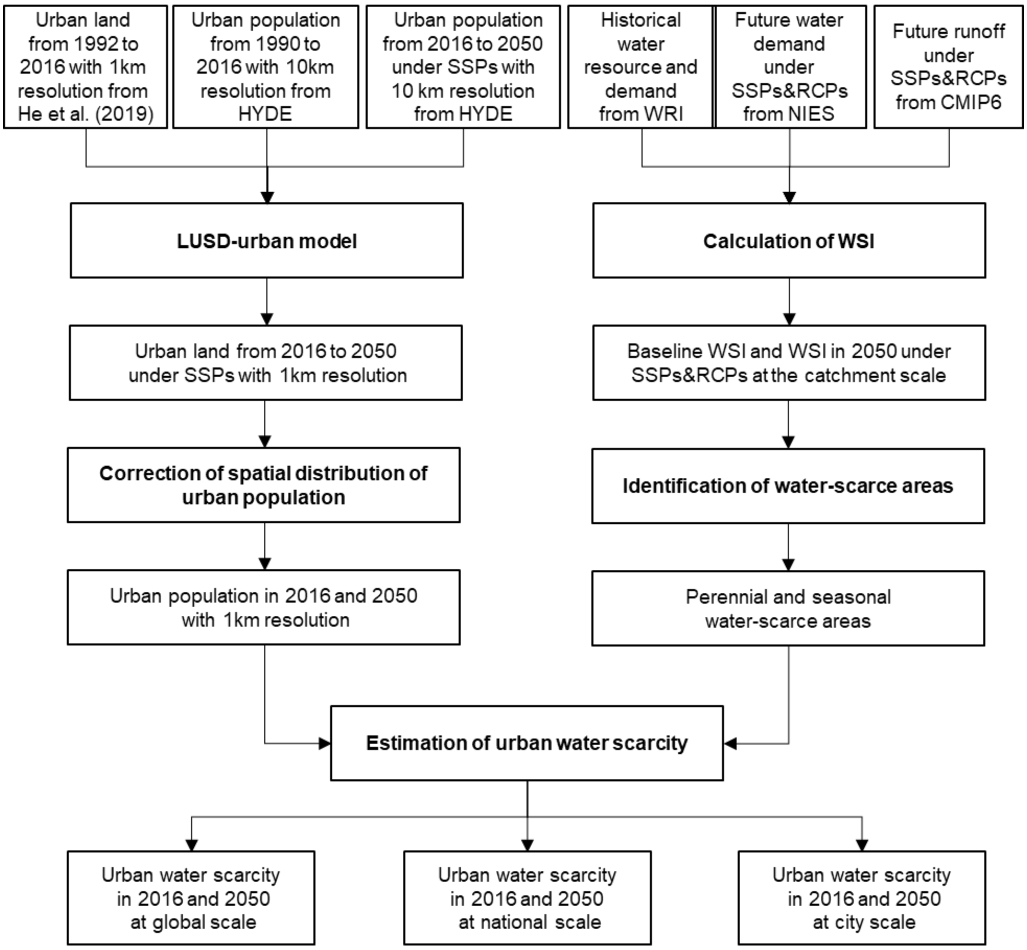

Supplementary Figure 1. Flow chart for estimating urban water scarcity. The bold text represents the key steps. The non-bold text represents input or output of these steps.

Note: HYDE = History Database of the Global Environment; SSPs = shared socioeconomic pathways; RCPs = representative concentration pathways; WRI = World Resource Institute; NIER = National Institute of Environmental Research; CMIP6 = Coupled Model Intercomparison Project Phase 6; LUSD = Land Use Scenario Dynamics; WSI = water stress index.

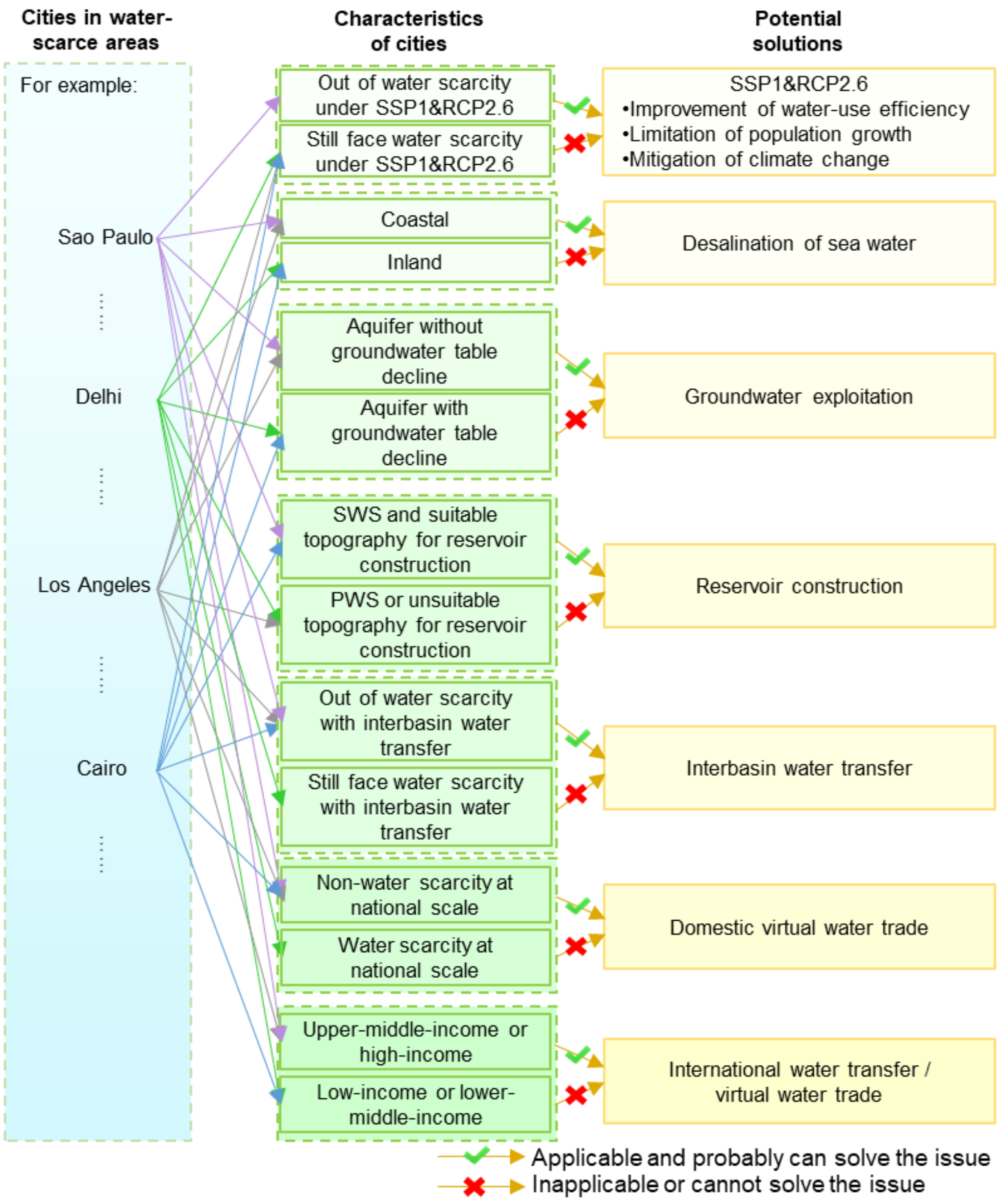

Supplementary Figure 2. Framework for selecting potential solutions. For each water-scarce city, the feasibility of potential solutions depends on its characteristics. For example, Sao Paulo can adopt desalination as it is a coastal city, Los Angeles can apply groundwater exploitation since it is located on an aquifer without groundwater table decline, Cairo can implement reservoir construction because it faces seasonal water scarcity and has suitable topography, Delhi is not likely to adopt the listed solutions due to its location and economic development level.

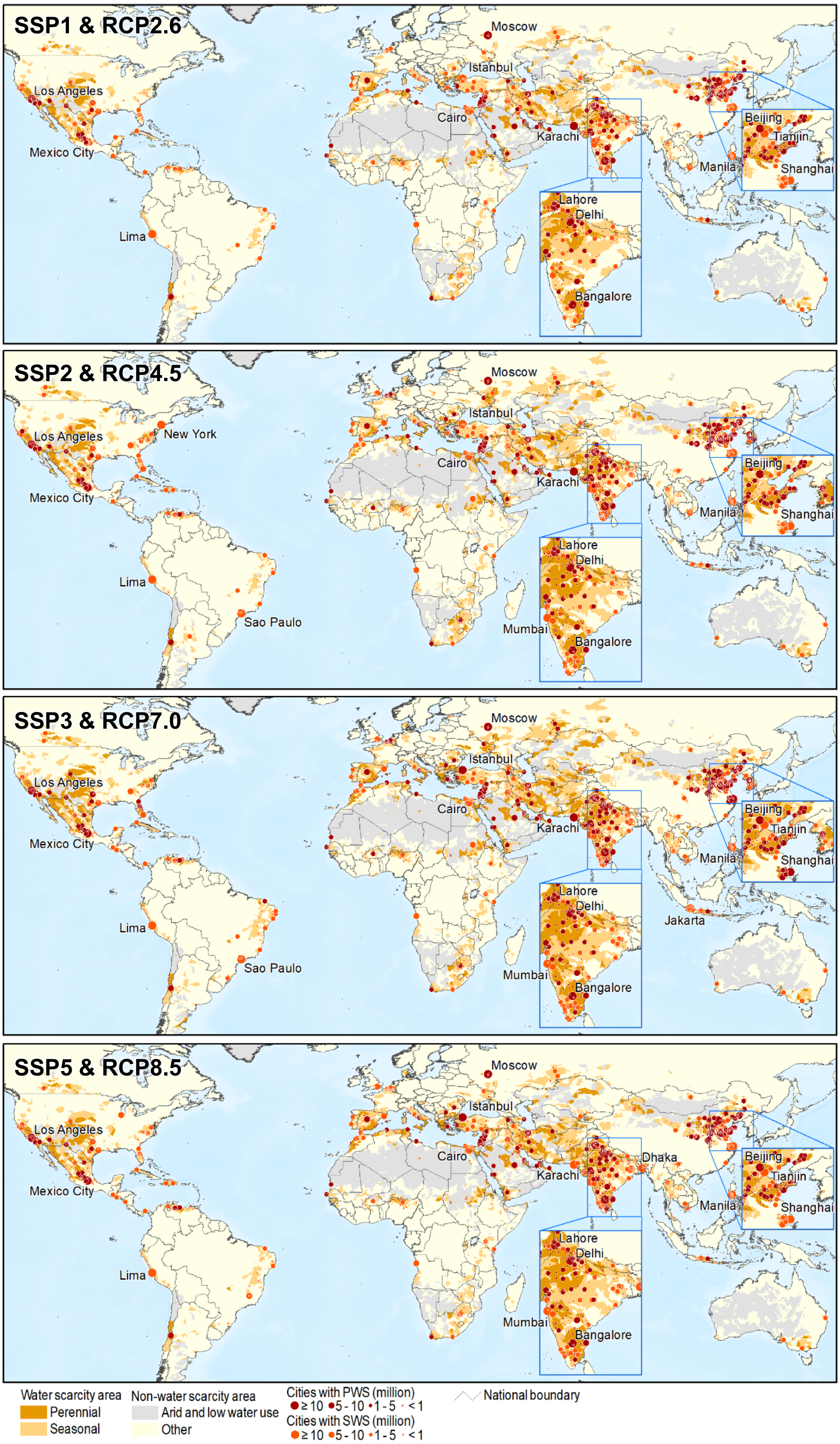

Supplementary Figure 3. Large cities subject to water scarcity in 2050 under four socio-economic and climate change scenarios.

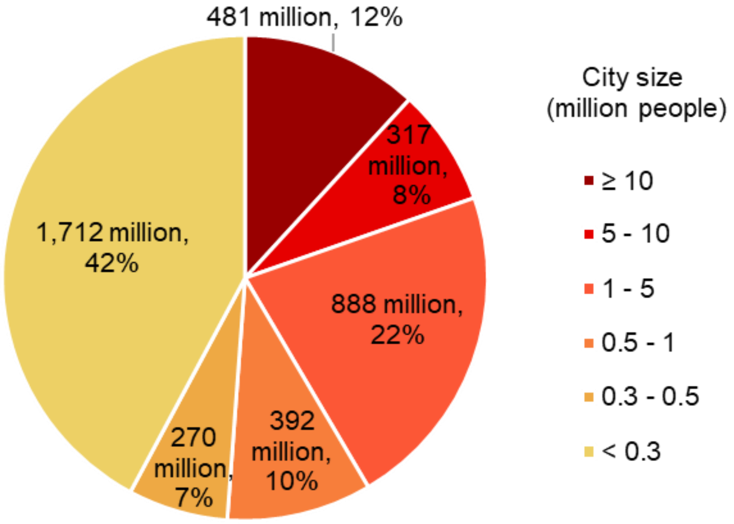

Supplementary Figure 4. Distribution of urban population in different city sizes in 2016.

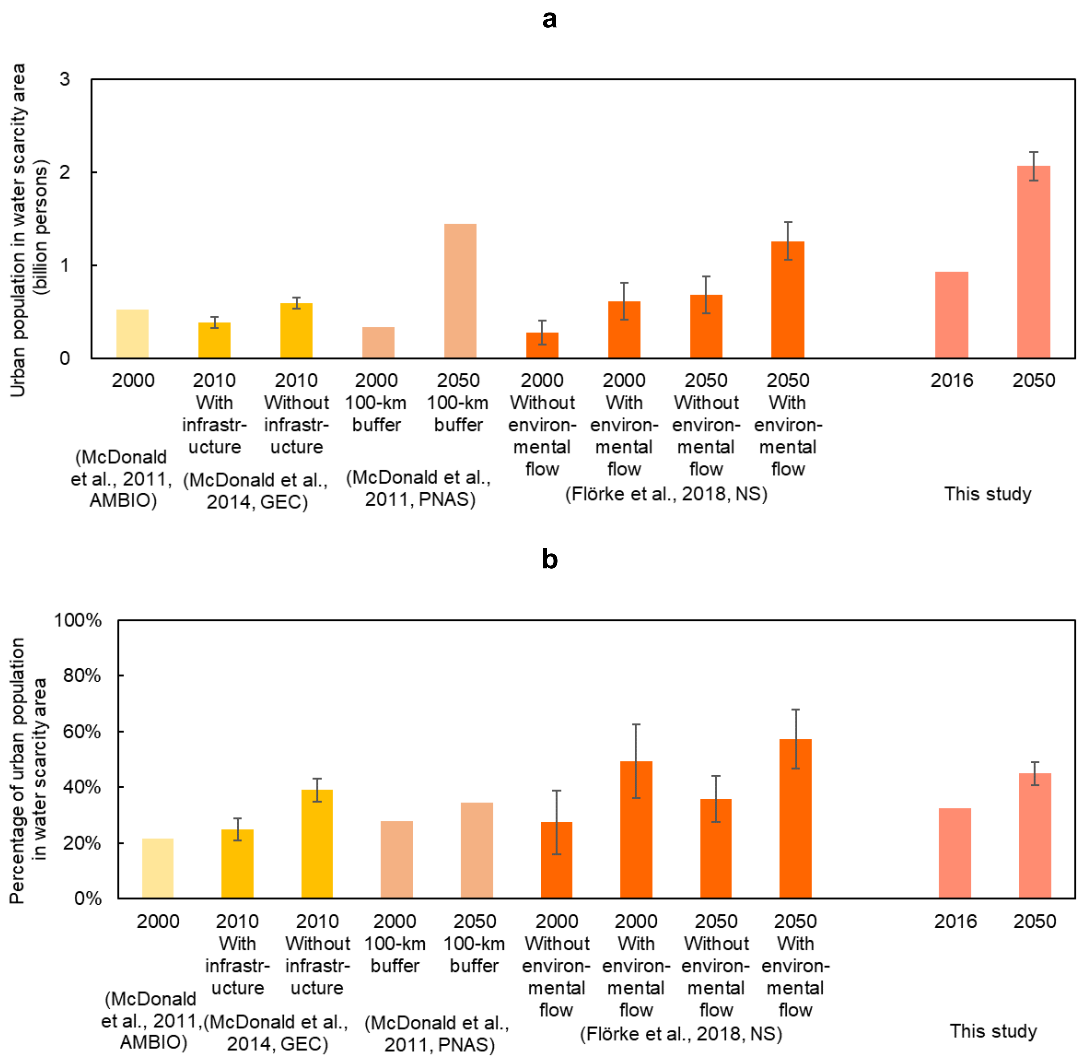

Supplementary Figure 5. Comparing urban exposure to water scarcity between our study and previous studies.

(a) Urban population in water scarcity area.

(b) Percentage of urban population in water scarcity area.

Note: The assessment results without buffer from McDonald et al. 2011, PNAS was not listed here since such results obviously overestimate the urban population exposed to water scarcity.

Supplementary Table 1. Details of the data sources used in this study.

Data	Time period	Spatial resolution (scale)	Scenario	Data source (Reference)	Link
Global urban population	1992-2016	10km	N/A	HYDE (Klein Goldewijk et al., 2010; 2017)	https://themasites.pbl.nl/tridion/en/themasites/hyde/download/index-2.html
	2050		SSPs		
Global urban land-use data	1992-2016	1km		(He et al., 2019)	https://doi.pangaea.de/10.1594/PANGAEA.892684
Global land-use/land-cover data	1992-2015	300m		ESA	http://maps.elie.ucl.ac.be/CCI/viewer/index.php
Global digital elevation model		1km		USGS	https://lta.cr.usgs.gov/
Locations of cities with population > 300,000				UN	https://population.un.org/wup/
Global coastline data				NOAA	https://www.ngdc.noaa.gov/mgg/shorelines
Global highway and railway data				CAS, REDCP	http://www.resdc.cn
Global river network dataset				(Schneider et al., 2017)	https://www.metis.upmc.fr/en/node/375
Global protected area dataset				ProtectedPlanet	http://www.protectedplanet.net
Global water resources/ withdrawal data (monthly available water resource, monthly water withdrawal and consumption for irrigation, livestock, industrial, and domestic sectors)	2014 (with a 10-year ordinary least square regression)	catchment		AQUEDUCT3.0 (Hofste et al., 2019)	https://www.wri.org/resources/data-sets/aqueduct-global-maps-30-data
Global water withdrawal data	2000	0.5 degree	N/A	NIER, Japan (Hanasaki et al., 2013a)	
	2050		SSP1&RCP2.6, SSP2&RCP4.5, SSP3&RCP6.0, SSP5&RCP8.5		
Global surface and subsurface runoff data	2005-2014	See Supplementary Table 2	N/A	CMIP6	https://esgf-node.llnl.gov/search/cmip6/
	2041-2050		SSP1&RCP2.6, SSP2&RCP4.5, SSP3&RCP7.0, SSP5&RCP8.5		
Global groundwater table data	1960-2014	5 min		AQUEDUCT3.0 (Hofste et al., 2019)	https://www.wri.org/resources/data-sets/aqueduct-global-maps-30-data
Global reservoir distribution data				GRanD V1.3 (Lehner, 2011)	http://globaldamwatch.org/grand/
Global pumped hydro atlas				RE100 research group of ANU	http://re100.eng.anu.edu.au
Interbasin water transfer data for global cities*	2014			City water map (Version 2.2) (McDonald et al., 2014)	https://knb.ecoinformatics.org/view/doi%3A10.5063%2FF1J67DWR
List of countries in four income categories	2020			World Bank	https://datahelpdesk.worldbank.org

Note: SSPs = shared socioeconomic pathways, HYDE = History Database of the Global Environment; ESA = European Space Agency; USGS = United States Geological Survey; UN = United Nations; NOAA = National Oceanic and Atmospheric Administration; CAS = Chinese Academy of Sciences; REDCP = Resource and Environmental Data Cloud Platform; NIER = National Institute of Environmental Research; CMIP6 = Coupled Model Intercomparison Project Phase 6; GRanD = Global Reservoir and Dam Database; ANU = Australian National University

*The data on "South-to-North Water Diversion" were updated according to Water Resources Bulletin in China.

Supplementary Table 2. Details of the global climate models used in this study.

Model	The ensembles used under different scenarios					Country	Institution	Resolution	Reference
	SSP1-RCP2.6	SPP2-RCP4.5	SSP3-RCP7.0	SSP5-RCP8.5	Sum				
MRI-ESM2-0	1	1	5	1	8	Japan	Meteorological Research Institute	1.125°× 1.125°	Yukimoto et al., 2019
MPI-ESM1-2-HR	1	2	9	1	13	Germany	Max Planck Institute for Meteorology	0.9375°× 0.9375°	Müller et al., 2018
INM-CM5-0	1	1	5	1	8	Russia	Institute of Numerical Mathematic	1.5°×2°	Volodin et al., 2017
GFDL-ESM4	1	1	1	1	4	America	National Oceanic and Atmospheric Administration	1°×1.25°	Krasting et al., 2018
EC-Earth3-Veg	4	4	4	4	16	Europe	European Centre for Medium-Range Weather Forecasts	0.703°× 0.703°	Wyser et al., 2019
EC-Earth3	1	11	1	1	14				
CESM2-WACCM	1	1	3	1	6	America	National Center for Atmospheric Research	0.9375°× 1.25°	Gettelman et al., 2019a; b
CESM2	2	3	2	2	9				
CAMS-CSM1-0	1	1	1	1	4	China	Chinese Academy of Meteorological Sciences	1.125°× 1.125°	Rong et al., 2019
BCC-CSM2-MR	1	1	1	1	4	China	National Climate Center	1.125°× 1.125°	Xin et al., 2019
**Total**	**14**	**26**	**32**	**14**	**86**				

Supplementary Table 3. Details of this study and comparison with previous studies.

	Object	Extent	Water scarcity assessment	Future water scarcity	Scenario framework for future assessment	Scenarios for future assessment
**This study**	Exposure of urban population	Global	Both water withdrawal and water availability were considered	Evaluated	IPCC-CMIP6	SSP1&RCP2.6; SSP2&RCP4.5; SSP3&RCP7.0; SSP5&RCP8.5
**McDonald et al., 2011, Ambio**	Exposure of urban population	Cities greater than 50,000 in population	Water withdrawal was not considered	Not evaluated	N/A	N/A
**McDonald et al., 2011, PNAS**	Exposure of urban population	Cities in developing countries with >100,000 people	Water withdrawal was not considered	Evaluated	MEA	Adaptive Management; Global Orchestration; Order from Strength; Technogarden
**McDonald et al., 2014, GEC**	Exposure of urban population	Urban agglomerations greater than 750,000 people	Both water withdrawal and water availability were considered	Not evaluated	N/A	N/A
**Padowski and Gorelick, 2014, ERL**	Exposure of cities	70 cities with populations exceeding 750, 000	Both water withdrawal and water availability were considered	Evaluated	Self-defined	Urban population growth and agricultural expansion under normal climate conditions
**Flörke et al., 2018, NS**	Exposure of urban population	482 cities containing 736 million people in 2018	Both water withdrawal and water availability were considered	Evaluated	IPCC-CMIP5	SSP2&RCP6.0
**Mekonnen and Hoekstra, 2016, SA**	Exposure of total population	Global	Both water withdrawal and water availability were considered	Not evaluated	N/A	N/A
**Veldkamp et al., 2017, NC**	Exposure of total population	Global	Both water withdrawal and water availability were considered	Not evaluated	N/A	N/A
**Veldkamp et al., 2016, ERL**	Exposure of total population	Global	Water withdrawal was not considered	Evaluated	IPCC-CMIP5	SSP1&RCP2.6; SSP3&RCP6.0; SSP5&RCP8.5
**Wada et al., 2014, NG**	Exposure of total population	Global	Both water withdrawal and water availability were considered	Evaluated	IPCC-CMIP5	SSP2&RCPs
**Schewe et al., 2014, PNAS**	Exposure of total population	Global	Water withdrawal was not considered	Evaluated	IPCC-CMIP5	SSP2&RCP8.5
**Hanasaki et al., 2013b, HESS**	Exposure of total population	Global	Both water withdrawal and water availability were considered	Evaluated	IPCC-CMIP5	SSP1&RCP2.6; SSP1&RCP6.0; SSP2&RCP4.5; SSP2&RCP8.5; SSP3&RCP6.0; SSP3&RCP8.5; SSP4&RCP2.6; SSP4&RCP6.0; SSP5&RCP6.0; SSP5&RCP8.5
**Arnell and Lloyd-Hughes, 2014, CC**	Exposure of total population	Global	Water withdrawal was not considered	Evaluated	IPCC-CMIP5	Five SSP×Four RCPs

Note: Green color denotes the merit on estimation of global urban water scarcity.

Supplementary Table 4. Results of this study and comparison with previous studies.

	Total/urban population	Criterion	Unit for assessment	Estimated period	Seasonal (billion)*	Perennial (billion)**	Total (billion)***	Percentage****
**This study**	Urban	WSI > 1	Catchment	2016<br>2050	0.57<br>0.90-1.65	0.36<br>0.48-0.91	0.93<br>1.69-2.37	32.53%<br>34.52%-51.34%
**McDonald et al., 2011, Ambio**	Urban	Aridity index < 0.5	Pixel	2000	N/A	N/A	0.52	21.70%
**McDonald et al., 2011, PNAS**	Urban	Water availability < 100 L per person per day	Urban extent	2000<br>2050	0.89<br>3.10	0.15<br>0.99	1.04<br>4.09	86.33%<br>97.45%
			100-km buffer	2000<br>2050	0.31<br>1.30	0.02<br>0.15	0.33<br>1.45	28.00%<br>34.40%
**McDonald et al., 2014, GEC**	Urban	WSI > 0.4	Urban agglomeration (Without infrastructure)	2010	N/A	N/A	0.59±0.06	39±4%
			Urban agglomeration (With infrastructure)		N/A	N/A	0.38±0.06	25±4%
**Padowski and Gorelick, 2014, ERL**	Number of cities	WSI > 1	Basin	2010<br>2040	N/A<br>N/A	N/A<br>N/A	25*****<br>31*****	36%<br>44%
**Flörke et al., 2018, NS**	Urban	WSI > 1	Subbasin (Without environmental flow requirements)	2000<br>2050	N/A<br>N/A	N/A<br>N/A	0.15-0.40<br>0.48-0.88	16.1%-38.9%<br>27.6%-44.0%
			Subbasin (With environmental flow requirements)	2000<br>2050	N/A<br>N/A	N/A<br>N/A	0.41-0.81<br>1.06-1.46	36.3%-62.5%<br>46.6%-67.8%
**Mekonnen and Hoekstra, 2016, SA**	Total	WSI > 1	Pixel (1km)	1996–2005	3.72	0.54	4.26	71.00%
**Veldkamp et al., 2017, NC**	Total	WSI > 1	Pixel (0.5°)	2010	N/A	N/A	2.59	37.60%
**Veldkamp et al., 2016, ERL**	Total	Water availability < 1700 m^3/capita per year	Water provinces	2000<br>2050	N/A<br>N/A	N/A<br>N/A	1.78<br>4.12-5.34	38.00%<br>56.5%-61.8%
**Wada et al., 2014, NG**	Total	WSI > 0.4	Watershed	2000<br>2050	N/A<br>N/A	N/A<br>N/A	1.9<br>3.68	31.70%<br>39.90%
**Schewe et al., 2014, PNAS**	Total	Water availability < 1000 m^3/capita per year	Country	2000<br>2050	N/A<br>N/A	N/A<br>N/A	0.18<br>2.04	3.00%<br>21.00%
**Hanasaki et al., 2013b, HESS**	Total	WSI ≥ 0.4	Pixel (0.5°)	2000<br>2050	N/A<br>N/A	N/A<br>N/A	1.72<br>1.73-3.42	28.29%<br>23%-39%
**Arnell and Lloyd-Hughes, 2014, CC**	Total	Water availability < 1000 m^3/capita per year	Watershed	2000<br>2050	N/A<br>N/A	N/A<br>N/A	1.56<br>3.29-4.77	25.59%<br>39.07%-46.65%

* The urban or total population facing seasonal water scarcity.
** The urban or total population facing perennial water scarcity.
*** The urban or total population facing water scarcity.
**** The percentage of urban or total population facing water scarcity.
***** The number of cities.

Supplementary Table 5. Potential solutions for addressing water scarcity of different cities*.

Country	City**	Population in 2016 (thousand)	Potential solutions							
			SSP1&RCP2.6***	Desalination of sea water	Groundwater exploitation	Reservoir construction	Interbasin water transfer	Domestic virtual water trade	International water transfer / virtual water trade	No solutions feasible
Algeria	El Djazair (Algiers)	2626	○	●	●	●	●	●	●	
Angola	Luanda	7265	○	●	●	○	●	●	○	
Argentina	Córdoba	1525	●	○	●	●	●	●	●	
Armenia	Yerevan	1074	○	○	●	○	●	●	●	
Australia	Melbourne	4541	○	●	○	●	●	●	●	
	Brisbane	2254	○	●	●	●	●	●	●	
	Perth	1926	○	●	○	●	●	●	●	
	Adelaide	1296	○	●	●	●	●	●	●	
Azerbaijan	Baku	2233	○	●	○	○	●	●	●	
Bangladesh	Dhaka	18234	●	●	○	○	●	●	○	
Belgium	Bruxelles-Brussel	2018	○	●	○	○	●	●	●	
	Antwerpen	1022	○	●	○	○	●	●	●	
Brazil	Sao Paulo	21136	●	●	●	●	●	●	●	
	Belo Horizonte	5826	●	○	●	●	●	●	●	
	Brasília	4267	○	○	●	●	●	●	●	
	Porto Alegre	4030	●	●	●	○	●	●	●	
	Recife	3905	●	●	●	●	●	●	●	
	Fortaleza	3858	○	●	●	○	●	●	●	
	Salvador	3647	●	●	●	●	○	●	●	
	Campinas	3100	●	○	●	●	●	●	●	
	Grande Vitória	1918	○	●	●	●	●	●	●	
	Joao Pessoa	1308	●	●	●	●	●	●	●	
	Maceió	1258	○	●	●	●	●	●	●	
Bulgaria	Sofia	1261	○	○	●	●	●	●	●	
Burkina Faso	Ouagadougou	2306	○	○	●	●	●	●	○	
Cambodia	Phnum Pénh (Phnom Penh)	1835	●	○	●	○	●	●	○	
Canada	Calgary	1398	○	○	●	●	●	●	●	
	Edmonton	1326	●	○	●	○	●	●	●	
Chile	Santiago	6575	○	○	●	○	●	●	●	
China	Shanghai	24163	○	●	○	●	●	●	●	
	Beijing	18812	○	○	○	○	●	●	●	
	Tianjin	12869	○	●	○	○	●	●	●	
	Xi'an, Shaanxi	6910	○	○	○	○	●	●	●	
	Hangzhou	6845	○	●	○	●	●	●	●	
	Shenyang	6636	○	○	●	○	●	●	●	
	Suzhou, Jiangsu	5652	○	●	○	●	●	●	●	
	Qingdao	5152	○	●	●	○	●	●	●	
	Dalian	4995	●	●	●	●	●	●	●	
	Ji'nan, Shandong	4755	○	○	●	●	●	●	●	
	Zhengzhou	4574	○	○	○	●	●	●	●	
	Changchun	4064	○	○	●	○	●	●	●	
	Kunming	4026	○	○	●	●	●	●	●	
	Shijiazhuang	3793	○	○	○	○	●	●	●	
	ürümqi (Wulumqi)	3670	○	○	●	●	●	●	●	
	Taiyuan, Shanxi	3567	○	○	●	○	●	●	●	
	Wuxi, Jiangsu	3038	○	●	○	●	●	●	●	
	Tangshan, Hebei	2878	○	●	○	○	●	●	●	
	Zibo	2475	○	○	○	●	●	●	●	
	Handan	2338	○	○	○	○	●	●	●	
	Weifang	2286	○	●	○	○	●	●	●	
	Yantai	2197	○	●	●	○	●	●	●	
	Huai'an	2197	○	○	○	●	●	●	●	
	Baotou	2006	●	○	●	○	●	●	●	
	Hohhot	1860	○	○	○	○	●	●	●	
	Baoding	1806	○	○	○	○	●	●	●	
	Linyi, Shandong	1754	○	●	○	○	●	●	●	
	Daqing	1670	○	○	●	○	●	●	●	
	Lianyungang	1591	○	●	○	○	●	●	●	
	Datong	1576	○	○	○	○	●	●	●	
	Anshan	1575	○	○	●	○	●	●	●	

	Putian	1528	○	●	●	●	●	●	●	
	Qiqihaer	1475	●	○	●	○	●	●	●	
	Jining, Shandong	1408	○	○	○	○	●	●	●	
	Yinchuan	1391	○	○	●	○	●	●	●	
	Xining	1367	○	○	●	●	●	●	●	
	Qinhuangdao	1333	○	●	●	●	●	●	●	
	Zhangjiakou	1302	○	○	●	○	●	●	●	
	Fushun, Liaoning	1296	○	○	●	○	●	●	●	
	Taian, Shandong	1245	○	○	●	○	●	●	●	
	Anyang	1203	○	○	○	○	●	●	●	
	Mianyang, Sichuan	1183	●	○	●	●	●	●	●	
	Zhanjiang	1178	○	●	●	●	●	●	●	
	Dongying	1136	○	●	○	○	●	●	●	
	Weihai	1131	○	●	●	○	●	●	●	
	Rizhao	1104	○	●	●	●	●	●	●	
	Benxi	1089	○	○	●	○	●	●	●	
	Maoming	1070	●	●	●	●	●	●	●	
	Yingkou	1064	○	●	●	○	●	●	●	
	Jinzhou	1058	○	●	●	●	●	●	●	
	Chifeng	1048	○	○	●	●	●	●	●	
	Zaozhuang	1041	○	○	●	○	●	●	●	
	Nanyang, Henan	1035	○	○	●	●	●	●	●	
	Baoji	1034	○	○	○	○	●	●	●	
	Pingdingshan, Henan	1029	○	○	○	○	●	●	●	
	Jiaxing	1026	○	●	○	○	●	●	●	
	Xinxiang	1010	○	○	○	○	●	●	●	
	Tengzhou	1003	○	○	○	○	●	●	●	
Cuba	La Habana (Havana)	2128	○	●	●	●	●	●	●	
Dem. People's Republic of Korea	P'yongyang	2993	●	●	●	●	●	●	○	
Dominican Republic	Santo Domingo	3019	●	●	●	●	○	●	●	
Egypt	Al-Qahirah (Cairo)	19230	○	○	○	●	●	●	○	
	Al-Iskandariyah (Alexandria)	4886	○	●	○	○	●	●	○	
El Salvador	San Salvador	1102	●	●	●	●	●	●	○	
Ethiopia	Addis Ababa	4040	●	○	●	●	●	●	○	
France	Lille	1046	○	●	●	○	●	●	●	
Georgia	Tbilisi	1078	●	○	○	●	●	●	●	
Greece	Athínai (Athens)	3159	○	●	●	●	●	●	●	
Guatemala	Ciudad de Guatemala (Guatemala City)	2775	○	●	●	●	●	●	●	
Haiti	Port-au-Prince	2503	●	●	●	●	○	●	○	
India	Delhi	26720	○	○	○	○	○	○	○	●
	Mumbai (Bombay)	19535	●	●	●	●	○	○	○	
	Bangalore	10557	○	○	●	○	○	○	○	
	Chennai (Madras)	9930	○	●	●	○	○	○	○	
	Hyderabad	8951	○	○	●	○	○	○	○	
	Ahmadabad	7295	○	●	○	○	○	○	○	
	Surat	5954	●	●	○	●	○	○	○	
	Pune (Poona)	5917	○	●	●	●	○	○	○	
	Jaipur	3520	○	○	●	●	○	○	○	
	Lucknow	3329	○	○	○	○	○	○	○	●
	Kanpur	3036	○	○	○	○	○	○	○	●
	Kozhikode (Calicut)	2810	●	●	●	●	○	○	○	
	Nagpur	2720	○	○	●	●	○	○	○	
	Kochi (Cochin)	2634	●	●	●	●	○	○	○	
	Indore	2627	○	○	●	●	○	○	○	
	Malappuram	2538	●	●	●	●	○	○	○	
	Coimbatore	2493	●	○	●	●	○	○	○	
	Thrissur	2488	○	●	●	●	○	○	○	
	Patna	2265	○	○	○	○	○	○	○	●
	Bhopal	2163	○	○	●	○	○	○	○	
	Thiruvananthapuram	2157	●	●	●	●	○	○	○	
	Vadodara	2027	○	●	○	○	○	○	○	
	Agra	2009	○	○	○	○	○	○	○	●
	Kannur	1928	●	●	●	●	○	○	○	
	Nashik	1837	○	○	●	●	○	○	○	
	Vijayawada	1781	●	●	●	●	○	○	○	
	Ludhiana	1753	○	○	○	●	○	○	○	
	Rajkot	1655	○	●	●	○	○	○	○	
	Madurai	1616	○	●	●	●	○	○	○	
	Meerut	1575	○	○	○	○	○	○	○	●
	Kollam	1494	●	●	●	●	○	○	○	

	Jamshedpur	1484	●	○	●	●	○	○	○	
	Srinagar	1442	○	○	●	○	○	○	○	
	Raipur	1400	○	○	●	○	○	○	○	
	Aurangabad	1393	○	○	●	●	○	○	○	
	Jabalpur	1371	○	○	●	○	○	○	○	
	Asansol	1349	●	○	●	○	○	○	○	
	Jodhpur	1321	○	○	●	○	○	○	○	
	Allahabad	1314	○	○	○	○	○	○	○	●
	Ranchi	1299	●	○	●	●	○	○	○	
	Amritsar	1292	○	○	○	○	○	○	○	●
	Dhanbad	1272	●	○	●	●	○	○	○	
	Gwalior	1255	○	○	○	○	○	○	○	●
	Tiruppur	1244	●	○	●	●	○	○	○	
	Kota	1210	○	○	●	●	○	○	○	
	Durg-Bhilainagar	1145	○	○	●	●	○	○	○	
	Bareilly	1134	○	○	○	○	○	○	○	●
	Mysore	1112	○	○	●	●	○	○	○	
	Tiruchirappalli	1103	○	●	●	●	○	○	○	
	Aligarh	1074	○	○	○	○	○	○	○	●
	Chandigarh	1070	○	○	○	○	○	○	○	●
	Moradabad	1056	○	○	○	○	○	○	○	●
	Hubli-Dharwad	1040	○	○	●	○	○	○	○	
	Bhubaneswar	1037	○	●	○	●	○	○	○	
	Salem	1020	●	○	●	●	○	○	○	
Indonesia	Jakarta	10287	●	●	●	●	●	●	○	
	Bekasi	2934	●	●	●	●	●	●	○	
	Surabaya	2868	○	●	●	○	●	●	○	
	Bandung	2502	●	●	●	●	●	●	○	
	Depok	2289	●	●	●	●	●	●	○	
	Tangerang	2110	●	●	●	●	●	●	○	
	Semarang	1737	○	●	●	●	●	●	○	
	Bogor	1072	●	●	●	●	●	●	○	
Iran (Islamic Republic of)	Tehran	8667	○	○	○	○	○	○	●	
	Mashhad	2989	○	○	●	○	○	○	●	
	Esfahan	1951	○	○	○	○	○	○	●	
	Karaj	1594	○	●	○	○	○	○	●	
	Shiraz	1560	○	○	●	○	○	○	●	
	Tabriz	1556	○	○	●	●	○	○	●	
	Qom	1196	○	○	○	●	○	○	●	
	Ahvaz	1181	○	●	●	○	○	○	●	
Iraq	Baghdad	6502	○	○	●	●	●	●	●	
	Al-Basrah (Basra)	1250	○	●	○	○	●	●	●	
Israel	Tel Aviv-Yafo (Tel Aviv-Jaffa)	3803	○	●	○	○	○	○	●	
	Hefa (Haifa)	1112	○	●	○	○	○	○	●	
Italy	Roma (Rome)	4145	○	●	●	●	●	●	●	
	Napoli (Naples)	2202	○	●	●	●	●	●	●	
Jordan	Amman	1872	○	●	●	○	○	○	●	
Kenya	Nairobi	4065	○	○	●	●	●	●	○	
	Mombasa	1139	○	●	●	○	●	●	○	
Kuwait	Al Kuwayt (Kuwait City)	2701	○	●	○	○	○	○	●	
Lebanon	Bayrut (Beirut)	2280	○	●	●	○	○	○	●	
Libya	Tarabulus (Tripoli)	1142	○	●	○	○	○	○	●	
Mauritania	Nouakchott	1105	○	●	●	○	●	●	○	
Mexico	Ciudad de México (Mexico City)	21420	○	○	○	●	●	●	●	
	Monterrey	4555	○	○	○	○	●	●	●	
	Puebla	3001	○	○	○	●	●	●	●	
	Toluca de Lerdo	2243	○	○	○	○	●	●	●	
	Tijuana	1978	○	●	●	○	●	●	●	
	León de los Aldamas	1736	○	○	○	○	●	●	●	
	Ciudad Juárez	1442	○	○	○	○	●	●	●	
	La Laguna	1370	○	○	○	●	●	●	●	
	Querétaro	1238	○	○	○	○	●	●	●	
	San Luis Potosí	1143	○	○	○	○	●	●	●	
	Mérida	1083	○	●	●	○	●	●	●	
	Mexicali	1044	○	●	○	○	○	●	●	
	Aguascalientes	1034	○	○	○	○	●	●	●	
	Cuernavaca	1013	○	○	○	●	●	●	●	
Mongolia	Ulaanbaatar	1415	○	○	●	●	●	●	○	
Morocco	Dar-el-Beida (Casablanca)	3623	○	●	●	●	●	●	○	
	Rabat	1812	○	●	●	●	●	●	○	
	Fès	1146	●	○	●	●	●	●	○	
	Tanger	1036	○	●	●	●	●	●	○	

Nepal	Kathmandu	1227	○	○	●	●	●	●	○	
Nicaragua	Managua	1034	●	●	●	●	●	●	○	
Nigeria	Kano	3661	○	○	●	●	●	●	○	
Oman	Masqat (Muscat)	1312	○	●	●	○	○	○	●	
Pakistan	Karachi	14651	○	●	●	○	○	○	○	
	Lahore	10808	○	○	○	○	○	○	○	●
	Faisalabad	3147	○	○	○	●	○	○	○	
	Gujranwala	1983	○	○	○	○	○	○	○	●
	Multan	1840	○	○	○	○	○	○	○	●
	Hyderabad	1707	●	○	●	●	○	○	○	
Panama	Ciudad de Panamá (Panama City)	1709	○	●	●	●	●	●	●	
Peru	Lima	10002	○	●	●	●	●	●	●	
Philippines	Manila	13064	○	●	●	●	●	●	○	
Portugal	Lisboa (Lisbon)	2898	○	●	●	●	●	●	●	
	Porto	1302	○	●	●	●	●	●	●	
Republic of Korea	Seoul	9919	●	●	●	○	●	●	●	
	Incheon	2711	●	●	●	●	●	●	●	
	Daegu	2236	●	●	●	●	●	●	●	
	Daejon	1543	●	●	●	●	●	●	●	
	Gwangju	1507	●	●	●	○	●	●	●	
	Suweon	1212	●	●	●	●	●	●	●	
Romania	Bucuresti (Bucharest)	1840	○	○	●	●	●	●	●	
Russian Federation	Moskva (Moscow)	12168	○	○	●	○	●	●	●	
	Yekaterinburg	1447	○	○	●	○	●	●	●	
	Chelyabinsk	1193	○	○	●	●	●	●	●	
	Omsk	1176	●	○	●	○	●	●	●	
	Voronezh	1034	●	○	●	○	●	●	●	
Saudi Arabia	Ar-Riyadh (Riyadh)	6440	○	○	○	○	○	○	●	
	Jiddah	4163	○	●	●	○	○	○	●	
	Makkah (Mecca)	1851	○	●	●	○	○	○	●	
	Al-Madinah (Medina)	1341	○	○	●	○	○	○	●	
	Ad-Dammam	1118	○	●	○	○	○	○	●	
Senegal	Dakar	2830	○	●	●	○	●	●	○	
South Africa	Johannesburg	5147	○	○	●	●	●	●	●	
	Cape Town	4208	○	●	○	○	●	●	●	
	Ekurhuleni	3559	○	○	●	●	●	●	●	
	Pretoria	2176	○	○	●	●	●	●	●	
	Port Elizabeth (Nelson Mandela Bay)	1198	○	●	●	●	●	●	●	
Spain	Madrid	6312	○	○	●	○	●	●	●	
	Barcelona	5348	●	●	●	○	●	●	●	
Sudan	Al-Khartum (Khartoum)	5260	○	○	●	●	●	●	○	
Syrian Arab Republic	Dimashq (Damascus)	2255	○	●	●	○	●	●	○	
	Halab (Aleppo)	1600	○	○	●	●	○	●	○	
	Hims (Homs)	1259	○	●	●	○	○	●	○	
Thailand	Chon Buri	1306	●	●	●	●	●	●	●	
	Chiang Mai	1090	○	○	●	●	●	●	●	
Tunisia	Tunis	2218	○	●	●	○	○	●	○	
Turkey	Istanbul	14332	○	●	●	●	●	●	●	
	Ankara	4727	○	○	○	●	●	●	●	
	Izmir	2885	○	●	○	○	●	●	●	
	Bursa	1849	○	●	○	●	●	●	●	
	Adana	1692	○	●	●	●	●	●	●	
	Gaziantep	1561	●	○	○	●	●	●	●	
	Konya	1215	○	○	○	●	●	●	●	
	Antalya	1116	○	●	○	●	●	●	●	
Ukraine	Kharkiv	1440	○	○	●	○	●	●	○	
United Arab Emirates	Dubayy (Dubai)	2523	○	●	○	○	○	○	●	
	Ash-Shariqah (Sharjah)	1367	○	●	○	○	○	○	●	
	Abu Zaby (Abu Dhabi)	1271	○	●	●	○	○	○	●	
United Kingdom	London	8788	●	●	●	○	●	●	●	
United States of America	New York-Newark	18705	●	●	●	●	●	●	●	
	Los Angeles-Long Beach-Santa Ana	12383	○	●	●	○	●	●	●	
	Chicago	8801	●	●	○	○	●	●	●	
	Miami	5902	●	●	●	●	●	●	●	
	Dallas-Fort Worth	5846	○	○	●	●	●	●	●	
	Houston	5807	○	●	○	●	●	●	●	
	Atlanta	5295	●	○	●	●	●	●	●	
	Phoenix-Mesa	4169	○	○	○	○	●	●	●	
	San Francisco-Oakland	3315	○	●	●	●	●	●	●	
	San Diego	3148	○	●	●	○	●	●	●	
	Denver-Aurora	2656	○	○	○	●	●	●	●	

	Riverside-San Bernardino	2258	○	●	●	○	●	●	●
	San Antonio	2096	○	○	●	●	●	●	●
	Sacramento	1969	○	○	○	●	●	●	●
	Orlando	1784	○	●	○	●	●	●	●
	Austin	1763	○	○	●	○	●	●	●
	San Jose	1749	○	●	●	●	●	●	●
	Charlotte	1706	●	○	●	○	●	●	●
	Raleigh	1203	●	○	●	●	●	●	●
	Jacksonville, Florida	1198	○	●	○	●	●	●	●
	Richmond	1049	●	●	○	●	●	●	●
Uzbekistan	Tashkent	2407	○	○	○	●	●	●	○
Venezuela (Bolivarian Republic of)	Caracas	2925	●	●	●	●	●	●	●
	Maracaibo	2093	○	●	●	●	●	●	●
	Valencia	1805	○	●	●	○	●	●	●
	Barquisimeto	1159	○	○	●	●	●	●	●
	Maracay	1148	○	●	●	○	●	●	●
Viet Nam	Thành Pho Ho Chí Minh (Ho Chi Minh City)	7605	○	●	●	●	●	●	○
	Can Tho	1265	●	●	●	●	●	●	○
Yemen	Sana'a'	2586	○	○	○	○	○	●	○

* The black dots denote that the solution is applicable and probably can solve the issue, the white dots denote that the solution is inapplicable or cannot solve the issue, the red dots denote that all the listed solutions are inapplicable or cannot solve the issue.

** The cities with population above 1 million in 2016, which would face water scarcity in 2050 under at least one scenario, were listed. Sort by country and population from largest to smallest.

*** Including improvement of water-use efficiency, limitation of population growth, and mitigation of climate change.

Supplementary Table 6. Spatial correlation on future urban expansion area between our estimates and existing datasets[*].

Scenarios	GPFULE[**]	GULFG[**]
SSP1	0.70^{***}	0.58^{***}
SSP2	0.72^{***}	0.54^{***}
SSP3	0.69^{***}	0.39^{***}
SSP4	0.62^{***}	0.53^{***}
SSP5	0.73^{***}	0.59^{***}

[*]The correlation coefficients were listed. The catchment was used as the basic unit to calculate urban expansion area and perform correlation analyses.

[**]GPFULE: global projections of future urban land expansion (Chen et al., 2020); GULFG: global 1/8-degree urban land fraction grids (Gao and O'Neill, 2020). According to data availability for different datasets, the urban expansion area between 2016 and 2050 in our estimates, the urban expansion area between 2015 and 2050 in GPFULE, and the urban expansion area between 2010 and 2050 in GULFG were used for correlation analyses.

[***]$P<0.001$

Supplementary Table 7. Comparison of urban population in cities with different sizes in 2016.

City size (population)	Total urban population (million)			Relative error	
	United Nations	HYDE	This study	HYDE	This study
Megacities (≥10 million)	480.91	243.83	283.80	-49.30%	-40.99%
Large cities (≥1 million)	1685.86	784.80	1061.87	-53.45%	-37.01%
All cities (≥0.3 million)	2364.58	987.87	1607.37	-58.22%	-32.02%

Supplementary Table 8. Impacts of interbasin water transfer on urban population facing water scarcity in 2016 (million persons).

	With interbasin water transfer			Without interbasin water transfer			Impacts of interbasin water transfer		
	**Peren.**	**Seas.**	**Total**	**Peren.**	**Seas.**	**Total**	**Peren.**	**Seas.**	**Total**
**Asia**	**268.0**	**340.8**	**608.8**	**273.6**	**340.6**	**614.3**	**-5.6**	**0.2**	**-5.5**
India	98.0	124.1	222.1	98.0	124.1	222.1	0	0	0
China	72.1	86.9	158.9	72.1	92.2	164.2	0	-5.3	-5.3
Pakistan	25.7	14.2	39.9	31.3	8.6	39.9	-5.6	5.6	0
Indonesia	0.0	29.0	29.0	0.0	29.0	29.0	0	0	0
Philippines	0.0	3.1	3.1	0.0	3.1	3.1	0	0	0
**Africa**	**13.8**	**67.1**	**80.9**	**13.8**	**67.1**	**80.9**	**0**	**0**	**0**
Nigeria	0.6	17.2	17.8	0.6	17.2	17.8	0	0	0
Egypt	1.7	0.0	1.7	1.7	0.0	1.7	0	0	0
**North America**	**45.7**	**62.8**	**108.5**	**60.9**	**52.7**	**113.6**	**-15.2**	**10.1**	**-5.1**
United States	24.0	26.9	50.9	24.0	32.1	56.1	0	-5.2	-5.2
Mexico	21.6	28.7	50.3	36.8	13.5	50.3	-15.2	15.2	0
**South America**	**7.7**	**28.8**	**36.5**	**7.7**	**28.8**	**36.5**	**0**	**0**	**0**
Brazil	0.1	7.7	7.7	0.1	7.7	7.7	0	0	0
**Europe**	**21.8**	**69.1**	**90.9**	**21.8**	**69.1**	**90.9**	**0**	**0**	**0**
**Oceania**	**0.4**	**2.5**	**2.8**	**0.4**	**2.5**	**2.8**	**0**	**0**	**0**
**World**	**359.3**	**573.4**	**932.7**	**380.1**	**563.3**	**943.3**	**-20.8**	**10.1**	**-10.6**

Supplementary References:

Arnell, N. W., & Lloyd-Hughes, B. (2013). The global-scale impacts of climate change on water resources and flooding under new climate and socio-economic scenarios. Climatic Change, 122, 127-140. doi: 10.1007/s10584-013-0948-4

Chen, G., Li, X., Liu, X., Chen, Y., Liang, X., Leng, J., Xu, X., Liao, W., Qiu, Y., Wu, Q., & Huang, K. (2020). Global projections of future urban land expansion under shared socioeconomic pathways. Nature Communications, 11, 537. doi: 10.1038/s41467-020-14386-x

Flörke, M., Schneider, C., & McDonald, R. I. (2018). Water competition between cities and agriculture driven by climate change and urban growth. Nature Sustainability, 1, 51-58. doi: 10.1038/s41893-017-0006-8

Gao, J., & O'Neill, B. C. (2020). Mapping global urban land for the 21st century with data-driven simulations and Shared Socioeconomic Pathways. Nat Commun, 11, 2302. doi: 10.1038/s41467-020-15788-7

Gettelman, A., Hannay, C., Bacmeister, J. T., Neale, R. B., Pendergrass, A. G., Danabasoglu, G., Lamarque, J. F., Fasullo, J. T., Bailey, D. A., Lawrence, D. M., & Mills, M. J. (2019a). High Climate Sensitivity in the Community Earth System Model Version 2 (CESM2). Geophysical Research Letters, 46, 8329-8337. doi: 10.1029/2019GL083978

Gettelman, A., Mills, M. J., Kinnison, D. E., Garcia, R. R., Smith, A. K., Marsh, D. R., Tilmes, S., Vitt, F., Bardeen, C. G., McInerny, J., Liu, H. L., Solomon, S. C., Polvani, L. M., Emmons, L. K., Lamarque, J. F., Richter, J. H., Glanville, A. S., Bacmeister, J. T., Phillips, A. S., Neale, R. B., Simpson, I. R., DuVivier, A. K., Hodzic, A., & Randel, W. J. (2019b). The Whole Atmosphere Community Climate Model Version 6 (WACCM6). Journal of Geophysical Research: Atmospheres, 124, 12380-12403. doi: 10.1029/2019JD030943

Hanasaki, N., Fujimori, S., Yamamoto, T., Yoshikawa, S., Masaki, Y., Hijioka, Y., Kainuma, M., Kanamori, Y., Masui, T., Takahashi, K., & Kanae, S. (2013a). A global water scarcity assessment under Shared Socio-economic Pathways - Part 1: Water use. Hydrology and Earth System Sciences, 17, 2375-2391. doi: 10.5194/hess-17-2375-2013

Hanasaki, N., Fujimori, S., Yamamoto, T., Yoshikawa, S., Masaki, Y., Hijioka, Y., Kainuma, M., Kanamori, Y., Masui, T., Takahashi, K., & Kanae, S. (2013b). A global water scarcity assessment under Shared Socio-economic Pathways - Part 2: Water availability and scarcity. Hydrology and Earth System Sciences, 17, 2393-2413. doi: 10.5194/hess-17-2393-2013

He, C., Okada, N., Zhang, Q., Shi, P., & Li, J. (2008). Modelling dynamic urban expansion processes incorporating a potential model with cellular automata. Landscape and Urban Planning, 86, 79-91. doi: 10.1016/j.landurbplan.2007.12.010

He, C., Zhao, Y., Huang, Q., Zhang, Q., & Zhang, D. (2015). Alternative future analysis for assessing the potential impact of climate change on urban landscape dynamics. Science of the Total Environment, 532, 48-60. doi: 10.1016/j.scitotenv.2015.05.103

He, C., Liu, Z., Gou, S., Zhang, Q., Zhang, J., & Xu, L. (2019). Detecting global urban expansion over the last three decades using a fully convolutional network. Environmental Research Letters, 14, 034008. doi: 10.1088/1748-9326/aaf936

Hofste, R. W., Kuzma, S., Walker, S., Sutanudjaja, E. H., Bierkens, M. F. P., Kuijper, M. J. M., Sanchez, M. F., Beek, R. V., Wada, Y., Rodríguez, S. G., & Reig, A. P. (2019). AQUEDUCT

3.0: Updated decision-relevant global water risk indicators. In. Washington, DC: World Resources Institute. doi: 10.46830/writn.18.00146

Klein Goldewijk, K., Beusen, A., & Janssen, P. (2010). Long-term dynamic modeling of global population and built-up area in a spatially explicit way-HYDE 3.1. The Holocene, 20, 565–573. doi: 10.1177/0959683609356587

Klein Goldewijk, K., Beusen, A., Doelman, J., & Stehfest, E. (2017). New anthropogenic land use estimates for the Holocene-HYDE 3.2. Earth System Science Data, 9, 927–953. doi: 10.5194/essd-9-927-2017

Krasting, J. P., John, J. G., Blanton, C., McHugh, C., Nikonov, S., Radhakrishnan, A., et al. (2018). NOAA-GFDL GFDL-ESM4 model output prepared for CMIP6 CMIP. Earth System Grid Federation. doi: 10.22033/ESGF/CMIP6.1407

Lehner, B., Liermann, C. R., Revenga, C., Vörösmarty, C., Fekete, B., Crouzet, P., Döll, P., Endejan, M., Frenken, K., Magome, J., Nilsson, C., Robertson, J. C., Rodel, R., Sindorf, N., & Wisser, D. (2011). High-resolution mapping of the world's reservoirs and dams for sustainable river-flow management. Frontiers in Ecology and the Environment, 9, 494-502. doi: 10.1890/100125

Liu, Z., Yang, Y., He, C., & Tu, M. (2019). Climate change will constrain the rapid urban expansion in drylands: A scenario analysis with the zoned Land Use Scenario Dynamics-urban model. Science of the Total Environment, 651, 2772-2786. doi: 10.1016/j.scitotenv.2018.10.177

McDonald, R. I., Douglas, I., Revenga, C., Hale, R., Grimm, N., Grönwall, J., & Fekete, B. (2011). Global Urban Growth and the Geography of Water Availability, Quality, and Delivery. Ambio, 40, 437-446. doi: 10.1007/s13280-011-0152-6

McDonald, R. I., Green, P., Balk, D., Fekete, B. M., Revenga, C., Todd, M., & Montgomery, M. (2011). Urban growth, climate change, and freshwater availability. Proceedings of the National Academy of Sciences of the United States of America, 108, 6312-6317. doi: 10.1073/pnas.1011615108

McDonald, R. I., Weber, K., Padowski, J., Flörke, M., Schneider, C., Green, P. A., Gleeson, T., Eckman, S., Lehner, B., Balk, D., Boucher, T., Grill, G., & Montgomery, M. (2014). Water on an urban planet: Urbanization and the reach of urban water infrastructure. Global Environmental Change, 27, 96-105. doi: 10.1016/j.gloenvcha.2014.04.022

Mekonnen, M. M., & Hoekstra, A. Y. (2016). Four billion people facing severe water scarcity. Sci Adv, 2, e1500323. doi: 10.1126/sciadv.1500323

Müller, W. A., Jungclaus, J. H., Mauritsen, T., Baehr, J., Bittner, M., Budich, R., Bunzel, F., Esch, M., Ghosh, R., Haak, H., Ilyina, T., Kleine, T., Kornblueh, L., Li, H., Modali, K., Notz, D., Pohlmann, H., Roeckner, E., Stemmler, I., Tian, F., & Marotzke, J. (2018). A Higher-resolution Version of the Max Planck Institute Earth System Model (MPI-ESM1.2-HR). Journal of Advances in Modeling Earth Systems, 10, 1383-1413. doi: 10.1029/2017MS001217

Padowski, J. C., & Gorelick, S. M. (2014). Global analysis of urban surface water supply vulnerability. Environmental Research Letters, 9, 104004. doi: 10.1088/1748-9326/9/10/104004

Rong, X., Li, J., Chen, H., et al. (2019). Introduction of CAMS-CSM model and its participation in CMIP6. Climate Change Research, 15 (5), 540-544. doi: 10.12006/j.issn.1673-1719.2019.186 (In Chinese)

Schewe, J., Heinke, J., Gerten, D., Haddeland, I., Arnell, N. W., Clark, D. B., Dankers, R., Eisner, S., Fekete, B.M., Colón-González, F.J., Gosling, S.N., Kim, H., Liu, X., Masaki, Y., Portmann, F.T., Satoh, Y., Stacke, T., Tang, Q., Wada, Y., Wisser, D., Albrecht, T., Frieler, K., Piontek, F., Warszawski, L., & Kabat, P. (2014). Multimodel assessment of water scarcity under climate change. Proceedings of the National Academy of Sciences, 111, 3245-3250. doi: 10.1073/pnas.1222460110

Schneider, A., Jost, A., Coulon, C., Silvestre, M., Théry, S., & Ducharne, A. (2017). Global-scale river network extraction based on high-resolution topography and constrained by lithology, climate, slope, and observed drainage density. Geophysical Research Letters, 44, 2773-2781. doi: 10.1002/2016gl071844

Veldkamp, T. I. E., Wada, Y., Aerts, J., Doll, P., Gosling, S. N., Liu, J., Masaki, Y., Oki, T., Ostberg, S., Pokhrel, Y., Satoh, Y., Kim, H., & Ward, P. J. (2017). Water scarcity hotspots travel downstream due to human interventions in the 20th and 21st century. Nature Communications, 8, 12. doi: 10.1038/ncomms15697

Veldkamp, T. I. E., Wada, Y., Aerts, J., & Ward, P. J. (2016). Towards a global water scarcity risk assessment framework: incorporation of probability distributions and hydro-climatic variability. Environmental Research Letters, 11, 12. doi: 10.1088/1748-9326/11/2/024006

Volodin, E. M., Mortikov, E. V., Kostrykin, S. V., Galin, V. Y., Lykossov, V. N., Gritsun, A. S., Diansky, N. A., Gusev, A. V., & Iakovlev, N. G. (2017). Simulation of the present-day climate with the climate model INMCM5. Climate Dynamics, 49, 3715-3734. doi: 10.1007/s00382-017-3539-7

Wada, Y., Gleeson, T., & Esnault, L. (2014). Wedge approach to water stress. Nature Geoscience, 7, 615-617. doi: 10.1038/ngeo2241

Wyser, K., van Noije, T., Yang, S., von Hardenberg, J., O'Donnell, D., & Döscher, R. (2019). On the increased climate sensitivity in the EC-Earth model from CMIP5 to CMIP6. Geosci. Model Dev. Discuss., 2019, 1-13. doi: 10.5194/gmd-2019-282

Xin X., Wu T., Zhang J., et al. (2019). Introduction of BCC models and its participation in CMIP6. Climate Change Research, 15 (5), 533-539. doi: 10.12006/j.issn.1673-1719.2019.039 (In Chinese)

Yukimoto, S., Kawai, H., Koshiro, T., Oshima, N., Yoshida, K., Urakawa, S., Tsujino, H., Deushi, M., Tanaka, T., Hosaka, M., Yabu, S., Yoshimura, H., Shindo, E., Mizuta, R., Obata, A., Adachi, Y., & Ishii, M. (2019). The Meteorological Research Institute Earth System Model Version 2.0, MRI-ESM2.0: Description and Basic Evaluation of the Physical Component. Journal of the Meteorological Society of Japan. Ser. II, 97, 931-965. doi: 10.2151/jmsj.2019-051
